# Anchoring geometry is a significant factor in determining the direction of kinesin-14 motility on microtubules

**DOI:** 10.1038/s41598-022-19589-4

**Published:** 2022-09-14

**Authors:** Masahiko Yamagishi, Rieko Sumiyoshi, Douglas R. Drummond, Junichiro Yajima

**Affiliations:** 1 Department of Life Sciences, Graduate School of Arts and Sciences, The University of Tokyo, 3-8-1 Komaba, Meguro-ku, Tokyo, 153-8902 Japan; 2 Centre for Promotion of International Education and Research, Faculty of Agriculture, Kyushu University, 744 Motooka, Nishi-ku, Fukuoka, 819-0395 Japan; 3 School of Interdisciplinary Science and Innovation, Kyushu University, 744 Motooka, Nishi-ku, Fukuoka, 819-0395 Japan; 4 Komaba Institute for Science, The University of Tokyo, 3-8-1 Komaba, Meguro-ku, Tokyo, 153-8902 Japan; 5 Research Center for Complex Systems Biology, The University of Tokyo, 3-8-1 Komaba, Meguro-ku, Tokyo, 153-8902 Japan; 6 Universal Biological Institute, The University of Tokyo, Bunkyo-ku, Tokyo, 113-0033 Japan

**Keywords:** Motility, Motor protein function, Motor protein tracks

## Abstract

Kinesin-14 microtubule-based motors have an N-terminal tail attaching the catalytic core to its load and usually move towards microtubule minus ends, whilst most other kinesins have a C-terminal tail and move towards plus ends. Loss of conserved sequences external to the motor domain causes kinesin-14 to switch to plus-end motility, showing that an N-terminal attachment is compatible with plus-end motility. However, there has been no systematic study on the role of attachment position in minus-end motility. We therefore examined the motility of monomeric kinesin-14s differing only in their attachment point. We find that a C-terminal attachment point causes kinesin-14s to become plus-end-directed, with microtubule corkscrewing rotation direction and pitch in motility assays similar to that of kinesin-1, suggesting that both C-kinesin kinesins-14 and N-kinesin kinesin-1 share a highly conserved catalytic core function with an intrinsic plus-end bias. Thus, an N-terminal attachment is one of the requirements for minus-end motility in kinesin-14.

## Introduction

The uni-directional movement of kinesin motor proteins along microtubules is important in many cellular processes in eukaryotes, including organelle transport and cell division. In general, most N-kinesins such as kinesins-1 to -10 and -12, in which the motor domain is located in the N-terminal part, move toward microtubule plus ends, whilst some C-kinesins such as kinesin-14, in which the motor domains are located at the C-terminal part, move toward the microtubule minus ends^1,2^. However, not all kinesins have such purely unidirectional motility and recently directional switching has been observed in some kinesins from yeasts and fungi. Some kinesin-5s, N-kinesins which are normally plus-end-directed, have the remarkable ability of also moving toward the minus-end of microtubules and switching directionality under various conditions^3–7^, whilst some kinesin-14 (C-kinesin, which are normally minus-end-directed) show context-dependent bidirectionality^8,9^. Despite the opposite endward directions of their longitudinal motilities, the catalytic core of N-kinesins such as kinesin-1 and C-kinesins such as kinesin-14 are remarkably similar in their 3D structure and amino acid sequences^1,10,11^. Furthermore N-kinesins such as the kinesins-1^12^, -2^13^, -3^14^, -5^15^, -6^16^, and -8^17^ and the C-kinesin kinesin-14^18,19^ also generate torque, which, together with their longitudinal motility, results in a corkscrew like translocation of microtubules gliding across an array of motors fixed to a surface. With the exception of the processive dimeric kinesin-1, which precisely tracks an individual protofilament in the microtubule^20^, plus-end-directed N-kinesins drive a left-handed corkscrew motion of the microtubule^12^, whilst minus-end-directed kinesin-14 Ncd drives a right-handed corkscrew motion^18^. This reversal of corkscrew handedness with direction suggests that the lateral torque generating component of kinesin motility is exactly the same in both types of motor (Supplementary Fig. 1)^12^.

**Figure 1:**
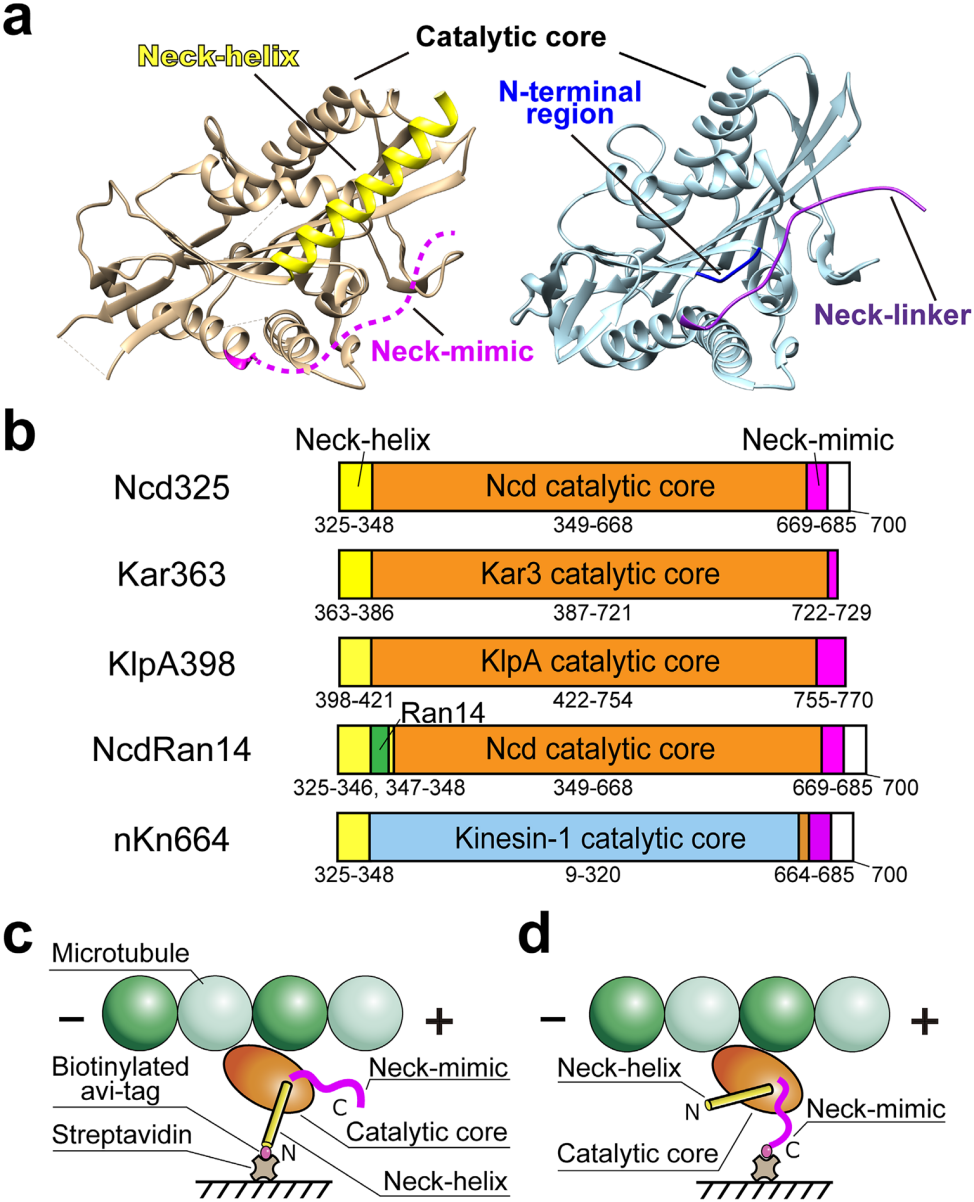
Observation of the directionality of N- or C-linked monomeric kinesin-14. (**a**) The 3D structure of Ncd (PDB: 5W3D)^43^ and kinesin-1 (PDB: 4HNA)^44^. The catalytic core (Ncd, orange; kinesin-1, cyan) has high structural homology among kinesins. The N-terminal neck-helix of Ncd (yellow) and cover-strand of kinesin-1 (blue) have little structural homology. The C-terminal neck-mimic of Ncd (pink) and neck-linker of kinesin-1 (purple) have little structural homology. (**b**) Monomeric constructs used in this study. All constructs (kinesin-14s, Ncd mutant, and kinesin-1—Ncd chimera) have biotinylated peptide (avi-tag) at either their N-terminus or C-terminus. NcdRan14 construct has an insertion of random 14 residues GESGAKQGEKGESG (green) between neck-helix and catalytic core, corresponding to the dimeric ncd-ran12 of the previous report^28^. nKn664 chimera is composed of Ncd K325-N348—RnKIF5C I9-K320—Ncd A664-K700^38^, corresponding to the dimeric chimera NcdKHC1 of the previous report^37^. (**c**,**d**) Scheme of a monomeric kinesin-14 anchored to the streptavidin-coated substrate via its N-terminus (**c**) and C-terminus (**d**), respectively.

In contrast to the similarity of their catalytic core structure and function, kinesin-1 and kinesin-14 have their own unique regions adjacent to the C- and N-terminus of their catalytic core, (Fig. 1a and Supplementary Fig. 2a,b). In kinesin-1, a C-terminal ~ 15 amino acid region called the neck-linker, which extends from the α6-helix in the catalytic core, undergoes conformational changes induced by changes in the kinesin nucleotide state^21,22^. The docking conformation of this neck-linker onto the catalytic core is believed to be the primary force-generating event for N-kinesins. Recently, an N-terminal β-strand called the cover-strand protruding from the kinesin-1 motor core has been implied to interact with the neck-linker forming a ‘cover-neck bundle’, which modulates force-generation along both the microtubule longitudinal^23,24^ and short lateral axes^25^. However, the neck-linker docking force-generating mechanism is not conserved in all N-kinesins, since some N-kinesins such as kinesins-6 and -10 lack typical neck-linker regions^26,27^. In contrast to the N-kinesins, the C-kinesin kinesins-14 possess a unique α-helical structure called the neck-helix joined directly to the N-terminus of the β1-sheet in the catalytic core that is highly conserved in all kinesin-14 members^28^. A rotational swing of the neck-helix has been hypothesized to be responsible for force-generation and minus-end directionality in kinesins-14, equivalent to the lever arm swing proposed for actin-based myosin motor proteins^29^, though the nucleotide state in which the neck-helix swings remains controversial^19,30–32^. In addition, kinesin-14 also contain a C-terminal short region called the neck-mimic that protrudes from the α6-helix. Although the neck-mimic has little similarity to the neck-linker of N-kinesins at the amino acid level, it does contain several basic and a hydrophobic amino acid that are highly conserved in kinesin-14s^33^. The neck-mimic was not detected in crystallographic or cryo-electron microscopy structures of wild-type kinesin-14s *Drosophila melanogaster* Ncd^30,34^ or *Saccharomyces cerevisiae* Kar3^35^, but was detected in the structure of kinesin-14 member KCBP (kinesin-like calmodulin binding protein), where the C-terminal region including the neck-mimic docked onto the catalytic core in the same way as the neck-linker in N-kinesins^36^. In Ncd with the single mutation T436S, the first three residues of the neck-mimic region were also shown to dock onto the catalytic core^31^. Biochemical and motility assays also indicate that the neck-mimic of Ncd can regulate Ncd binding affinity to microtubules and minus-end-directed motility^33^. These studies suggest that the C-terminal neck-mimic region in C-kinesins may also be involved in force generation for minus-end-directed motility in a similar way to the neck-linker of N-kinesins for plus-end-directed motility.

Chimeras fusing portions of the C-kinesin kinesin-14 Ncd with parts of a kinesin-1 have been used to identify which of the sequence features determine kinesin directionality. Ncd neck-helix and neck-mimic regions along with 5 amino acids (AASVN) of the catalytic core were fused to the N- and C-termini respectively, of an N-kinesin kinesin-1 catalytic motor core with the construct anchored via its N-terminus to the substrate surface (Supplementary Figs. 2 and 3). Both the dimeric NcdKHC1^37^ and monomeric nKn664^38^ versions of the construct have microtubule minus-end directionality, reversing the normal plus-end polarity of kinesin-1 movement (Supplementary Figs. 2c,d and 3). However, if mutations were present in the N-terminal neck-helix—catalytic core junction (NcdKHC5), directionality did not switch and the NcdKHC5 retained the plus-end directionality of kinesin-1^37^ (Supplementary Figs. 2e and 3). Sablin et al*.* also found that mutation of the conserved residues of the neck-helix in a dimeric Ncd (ncd-ran12) construct, in which the neck-helix was mutated by randomizing 12 residues and the N-terminus was anchored to the surface in a motility assay, was a slow plus-end-directed motor, reversing the normal minus-end directionality of Ncd^28^ (Supplementary Figs. 2j and 3). Thus, both the neck-helix and its linkage to the motor catalytic are important factors in determining minus-end directionality.

Structural analysis of a minus-end directed chimeric monomer construct (nKn664) showed that the nucleotide dependent conformational changes of the neck-mimic, which is located close to the switch II cluster, and its association with the N-terminal neck-helix, coupled ATP hydrolysis to the neck-helix rotational swing^38^ (Supplementary Figs. 2d and 3). However, nKn669, where the kinesin-14’s five amino acids (AASVN) at the catalytic core—C-terminal neck-mimic junction were replaced with those of kinesin-1 (GQRAK), lacked the neck-mimic interaction with the neck-helix—catalytic core junction seen in nKn664. In motility assays, the directionality of nKn669 did not switch, so that nKn669 retained the plus-end directionality of kinesin-1 (Supplementary Figs. 2f and 3). Taken together, these results suggest that proper functioning and interaction of both the Ncd neck-helix and neck-mimic are required to reverse the plus-end-directed polarity of kinesin-1 catalytic core movement to become minus-end directed. Also, since both the plus-end-directed mutant (ncd-ran12) and chimeras (NcdKHC5 and nKn669) lack the kinesin-1 neck-cover strand and kinesin-1 neck-linker, the kinesin-1 catalytic core itself may contain plus-end polarity determinants as suggested previously^28,39^, at least in the absence of functional minus end determinants.

Other studies have shown that complementary dimeric chimeras of ncd-Nkin^40^ and NK-1^41^ containing either the Ncd neck-helix or the kinesin-1 cover-strand followed by the Ncd catalytic core fused to part of the kinesin-1’s α6 (GMRAK or K) plus a C-terminal kinesin-1 neck-linker/stalk region, both moved towards the microtubule plus-end, reversing the minus-end polarity of Ncd movement (Supplementary Figs. 2g,h and 3). Both chimeras were anchored to the surface via their C-terminal kinesin-1 stalk in the motility assays. However, if dimeric chimera NcdKHC6, consisting of the Ncd stalk/neck-helix region and Ncd catalytic motor core region fused to part of kinesin-1’s α6 (GRRAK) plus the neck-linker region of kinesin-1, was anchored to the surface via its N-terminal neck-helix, it retained the minus-end polarity of Ncd movement^37^ (Supplementary Figs. 2i and 3). This suggested that the combination of part of kinesin-1’s α6 (GRRAK) and the neck-linker of kinesin-1 can substitute for the combination of part of Ncd’s α6 (AASVN) and the neck-mimic of Ncd sufficiently well to generate minus-end-directed motility. However, it is still unclear if the Ncd neck-mimic can substitute for the kinesin-1 neck-linker sufficiently well to replace it for plus endward motility if the Ncd neck-mimic is used to anchor the catalytic core via a C-terminal surface attachment, or if this is a specific function of the conserved neck-linker sequence. So, the functional equivalence of the neck-linker and neck-mimic remain unclear, and in particular the ability of the neck-mimic to act as an “effective” neck-linker to support plus-end-directed motility when the neck-mimic is anchored directly to the substrate surface has never been examined.

The most striking structural feature of the kinesin-14 family of minus-end-directed motors is that they are all C-terminal motors, whilst the plus-end-directed kinesins-1 are N-terminal. Furthermore, in the direction reversal studies using chimeras minus-end direction was only observed in N-terminal anchored constructs (see Supplementary Figs. 2c,d,i and 3 and Refs.^37,38^), whilst plus-end motility occurred in both N- and C-terminal anchored constructs (see Supplementary Figs. 2e,f,g,h,j and 3 and Refs.^28,37,38,40,41^), which has sometimes led to the assumption that the role of anchoring geometry is obvious. However, in these studies the anchoring geometry effect was never studied independently of the sequences surrounding the catalytic motor core. So, all of the N-terminal anchored constructs with minus-end-directed polarity contained neck-helix, whilst C-terminal anchored constructs contained neck-linker sequences. Recent observations of reversal in some native kinesins have also raised questions on the role of anchoring in direction determination. The bidirectional motility of double-headed kinesin-14s KlpA^9^ and Kar3^8^ show that anchoring of the N-terminus is compatible with both plus- and minus-end-directed motility in the native C-kinesins, whilst native double-headed or quadruple-headed N-kinesin kinesin-5s^3,4^ can have both minus as well as plus-end-directed motility showing that other kinesin geometries are potentially compatible with minus-end motility. For at least some kinesin-5s where dimeric or tetrameric constructs were bidirectional, monomeric constructs showed only robust, plus-end-directed motility^5,42^, suggesting a switching mechanism requiring at least two kinesin heads. The C-terminal kinesin-14 KlpA double-headed construct contains an additional microtubule binding site in the stalk^9^. Direction is determined by the motor and stalk either binding to the same or different microtubules, suggesting a potential relationship to anchoring geometry since on removal of the second microtubule binding site the KlpA becomes only minus end directed. Thus the role of anchoring geometry, and specifically a requirement for N-terminal anchoring to generate minus-end motility, remains unclear in native kinesin-14s.

In this study we have investigated the role of anchoring geometry in determining native single-headed kinesin-14 direction and the ability of the kinesin-14 neck-mimic to substitute for the neck-linker in plus-end-directed motility when the neck-mimic is anchored to the substrate. To separate the role of anchoring geometry from other factors, we have used monomeric constructs to distinguish between activities inherent in the single heads from the role of the relative interactions of the heads in the dimer. Minimal constructs comprising of only the catalytic core, neck-helix and neck-mimic from three different kinesin-14s were created with either an N- or C-terminal affinity tag to anchor the kinesin. We examined the direction of microtubule gliding and corkscrewing motion in microtubule motility assays. N-terminal anchored kinesin-14 monomeric constructs were minus-end directed as reported previously^30^. However, when C-kinesin kinesin-14s are attached to a substrate surface via their C-terminal neck-mimic region, they reverse direction to become plus-endward with a left-handed corkscrew motion. The handedness and pitch of microtubule corkscrew are the same as is observed with genuine N-kinesin monomeric kinesin-1 and kinesin-5 driven-microtubule corkscrewing motility^15^, indicating a strong conservation of catalytic core activity between N-kinesin and C-kinesins. These results show that in addition to the previously identified neck-helix and neck-mimic regions^37,38^, an N-terminal anchoring attachment is required to generate microtubule minus-end-directed motility. Furthermore, this effect occurs in monomeric kinesins suggesting the mechanism does not depend upon the relative head geometries of dimers and explains the conserved C-kinesin configuration of the kinesin-14 family.

## Results

### C-linked monomeric kinesin-14s glide microtubules with plus-end-directed motility

Native kinesin-14 motor proteins share a common capacity for microtubule minus-end directionality and a distinctive structure with the kinesin tail linked to the N-terminus of the conserved catalytic core via a neck-helix structure, whilst the C-terminus has a neck-mimic region located where the neck-linker joins the motor domain to the C-terminal stalk and tail domains of other N-kinesins (Fig. 1a). Although the neck-helix and neck-mimic are required for minus-end motility^37,38^, the role of N-terminal anchoring in determining the directionality of kinesin-14 motors remains unclear. To examine the role of substrate attachment in determining directionality, we created a series of constructs comprising a minimal motor domain from a kinesin-14 with the same anchoring tag at either their N- or C-terminus. To exclude motor head interactions in dimeric constructs, monomeric kinesin-14 minimal motor domains from either *Drosophila melanogaster* Ncd, 325–700 a.a.^18^; *Saccharomyces cerevisiae* Kar3, 363–729 a.a.^45^; or *Aspergillus nidulans* KlpA, 398–770 a.a.^9^ were fused to a biotinylated peptide (avi-tag)^46^ at either their N-terminus (BP-Ncd325, BP-Kar363, BP-KlpA398) or C-terminus (Ncd325-BP, Kar363-BP, KlpA398-BP) (Fig. 1b and Supplementary Fig. 3). These constructs allowed us to bind the same kinesin-14 monomers to a streptavidin-coated substrate either via their neck-helix at the N-terminus of the motor domain (Fig. 1c), or via the neck-mimic at the C-terminus (Fig. 1d). With N-terminal attachment, the neck-helix, which has been proposed to swing to create the minus-ended force, is attached to the surface and can transmit any force generated^30^, whilst with C-terminal attachment, the N-terminal neck-helix is free in solution and any swinging motion of the neck-helix would not be expected to directly exert a force on the motor.

**Figure 2:**
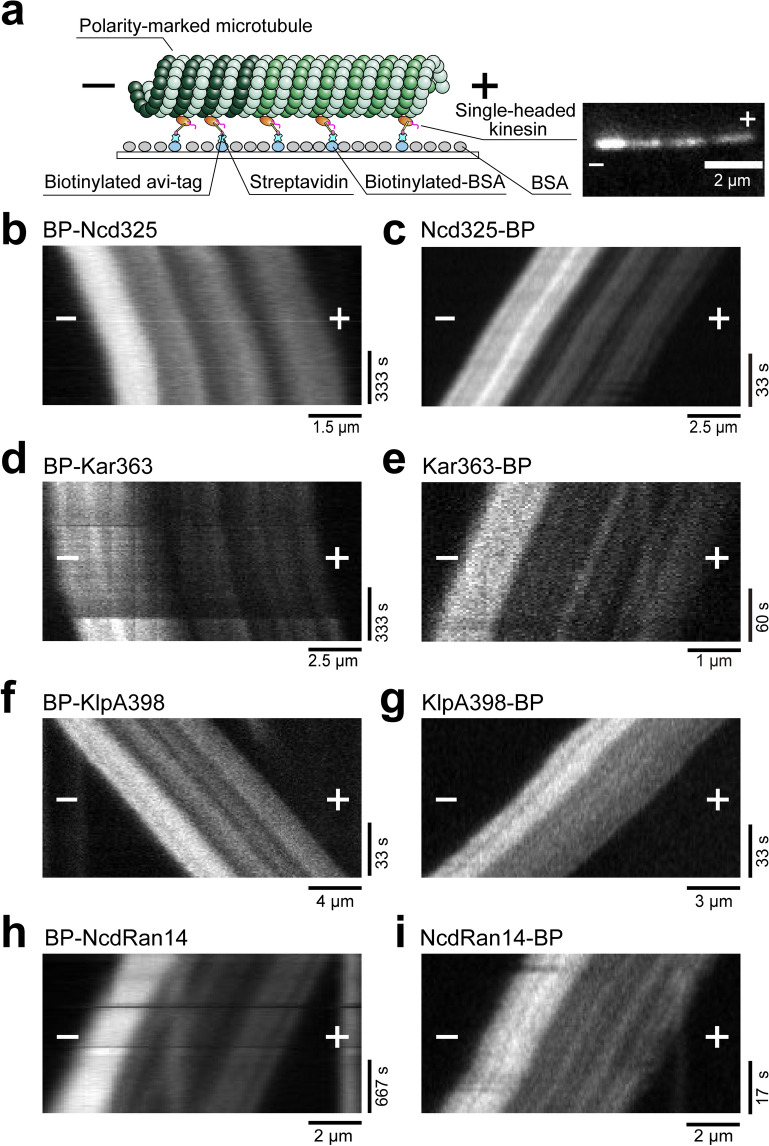
Polarity-marked microtubule gliding assays driven by N- or C-linked monomeric kinesins. (**a**) Scheme of the in vitro polarity-marked microtubule gliding assay. Single-headed kinesins fused to a biotinylated tag (avi-tag) are anchored to biotinylated BSA via biotin-streptavidin linkage. (**b**–**g**) Typical kymographs of polarity-marked microtubule gliding driven by N-linked monomeric Ncd (**b**), Kar3 (**d**), KlpA (**f**) and C-linked monomeric Ncd (**c**), Kar3 (**e**), KlpA (**g**) are shown. N-linked monomeric kinesin-14s glide microtubules with their dim plus-ends leading, indicating minus-end-directed motor activity, whereas C-linked monomeric kinesin-14s glide with their bright minus-ends leading, indicating plus-end-directed motor activity. (**h**,**i**) Typical kymographs of polarity-marked microtubule gliding driven by Ncd mutant NcdRan14 are shown. Both N- (**h**) and C- (**i**) linked monomeric kinesins glide microtubules with their bright minus-ends leading, indicating plus-end-directed motor activity. The plus (+) and minus (−) signs refer to the plus-end and minus-end of the microtubules, respectively.

We first assayed the longitudinal directionality of the N-linked and C-linked kinesin-14 monomeric constructs (Ncd325, Kar363 and KlpA398) using an in vitro polarity-marked microtubule gliding assay^38,47^ (Fig. 2). Lawns of either N-linked kinesin-14 Ncd325, Kar363 or KlpA398 constructs drove microtubule gliding with the dim plus-ends leading, indicating a minus-end-directed motor activity (Fig. 2b,d,f and Supplementary Movie 1), which is consistent with previous reports^9,30,45^. Remarkably, we found that lawns of C-linked kinesin-14 Ncd325, Kar363 or KlpA398 constructs drove microtubule gliding with the bright minus-ends leading, indicating a plus-end-directed motor activity, similar to kinesin-1 (Fig. 2c,e,g and Supplementary Movie 1), reversing their normal minus-end-directed longitudinal directionality.

### C-linked kinesin-14 monomers move QDs to the plus-end of microtubules

**Figure 3:**
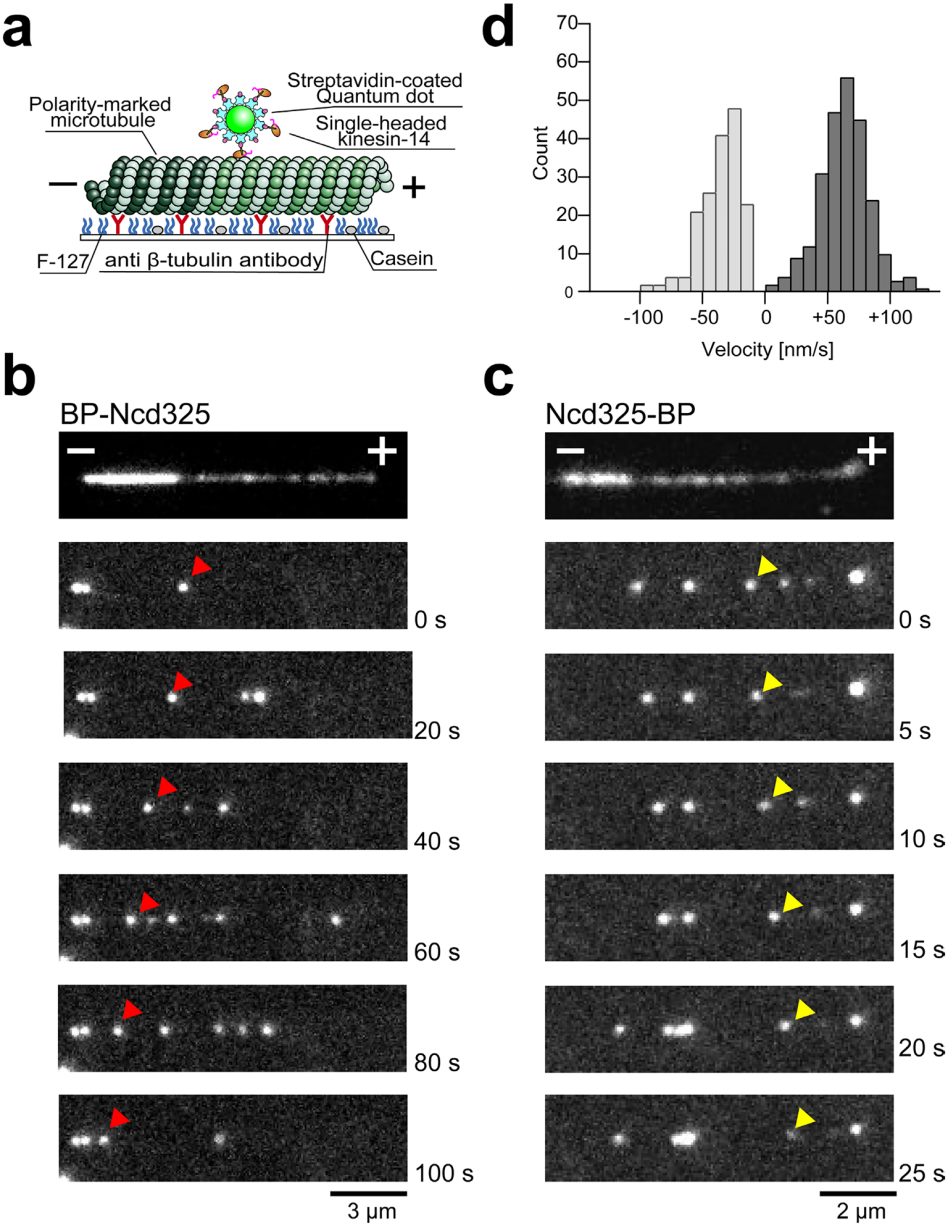
QD assay of N- or C-linked monomeric Ncd325. (**a**) Scheme of the quantum dot (QD) assay. Streptavidin-coated QDs, which are linked to avi-tag fused Ncds via either an N- or C-terminal avi-tag and biotin-streptavidin linkage, move on polarity-marked microtubules. (**b**,**c**) Sequential images of QDs moving on polarity-marked microtubules. QD-BP-Ncd325 (red arrow head, **b**) move towards the bright minus-end of the microtubule and Ncd325-BP-QD (yellow arrow head, **c**) towards the dim plus-end of the microtubule. (**d**) A histogram of motility velocities of a QD coated with N-linked monomeric Ncd (gray) and C-linked monomeric Ncd (black). Mean velocities for QD-BP-Ncd325 and Ncd325-BP-QD are − 37 ± 16 nm s^−1^ (mean ± SD, *n* = 171) and + 63 ± 20 nm s^−1^ (mean ± SD, *n* = 248), respectively. The plus (+) and minus (−) signs refer to the plus-end and minus-end of the microtubule.

To determine if the configuration of the microtubule gliding assay, with the kinesins stationary and anchored to the chamber substrate, influenced the kinesin motion we also assayed the directionality of N-linked and C-linked kinesin-14 monomeric constructs (Ncd325, Kar363 and KlpA398) by attaching multiple motors to quantum dots (QDs), and allowing the motors and QDs to move along, polarity-marked microtubules immobilized on the substrate (Fig. 3a). QDs carrying N-linked kinesin-14s (Ncd325, Kar363 and KlpA398) again moved towards microtubule minus-ends, whilst C-linked kinesin-14s (Ncd325) still moved towards plus-ends (Fig. 3b–d and Table 2 and Supplementary Movie 2). C-linked Kar363 and KlpA398 did not show stable unidirectional movement along microtubules, probably because of their extremely low mechanical processivity. These data clearly indicate that directionality in kinesin-14 monomers does not depend on the assay geometry or the particular forces acting in microtubule gliding assays, but instead depends on which kinesin-14 motor domain terminus is tethered to the substrate. For all three kinesin-14 catalytic cores tested coupling via the N-terminal neck-helix produces minus-end directionality as expected, whilst coupling via the C-terminal neck-mimic reverses the direction and generates plus-end-directed motility.

### Dysfunction of the precise neck-helix coupling causes the N-anchored Ncd monomer to generate slow plus-end-directed movement

Our results suggest that anchoring of the motor domain via the N-terminus is critical for minus-end-directed motility in kinesin-14 motors. Sablin et al*.* have reported that a mutant ncd-ran12, where 12 amino acids of the neck-helix between the N-terminus of the motor and the tail of a dimeric Ncd construct are replaced by 12 randomised amino acids, changes its directionality from minus-end to a slow plus-end-directed motility^28^. However, it was unclear if this arose from changes in the interactions of the heads in the dimer or from changes in the function of the individual heads. For example, the 12 amino acid replacements may have permitted the unattached motor domain of the two-headed Ncd to tilt towards the microtubule plus-end, resulting in the plus-end directionality. We therefore created two similar monomeric constructs NcdRan14 by inserting an additional 14 random amino acids between the neck-helix and catalytic core of our monomeric constructs (Fig. 1b) with the avi-tag attached at either the N- or C-terminus of each construct (Fig. 1c,d and Supplementary Fig. 3). In microtubule and QD motility assays both C-linked and N-linked monomeric NcdRan14 displayed plus-end-directed motility (Fig. 2h,i and Supplementary Movies 1 and 2). This demonstrates that despite the construct still being anchored to the substrate via its N-terminus, this was insufficient, and that direct coupling of the neck-helix to the motor domain is critical to generate minus-end directionality. In the absence of the precise neck-helix coupling the monomeric Ncd generates slow plus-end-directed movement, similar to the corresponding dimeric mutant, ncd-ran12^28^, and shows that this effect is intrinsic to the monomeric head and does not depend on the presence of the second head in the dimer.

### C-linked configuration can amplify the plus-end-directed bias of the motor core more efficiently

The 5 nm s^−1^ velocity of microtubule gliding driven by our N-linked monomeric Ncd minus-end-directed construct (Table 1) is similar to the 4 nm s^−1^ previously reported for a similar monomeric Ncd construct^30^, suggesting that the velocity of the N-linked constructs in our assay was not being inhibited by the anchoring tag used in our constructs. The microtubule gliding assay (Table 1 and Fig. 2 and Supplementary Fig. 4) and QD motility assay (Table 2 and Fig. 3 and Supplementary Fig. 5) also showed a consistent trend with speeds of C-linked kinesin-14s 1.5–150 times faster compared to speeds of N-linked kinesin-14s. In particular, C-linked NcdRan14 was much faster than N-linked NcdRan14 in both assays. Both constructs have identical motor domains and plus-ended directionality, suggesting these differences may not simply arise from variations in the numbers of motors working in the teams bound to the QDs. Our results extend earlier work^31,33,39,48^ by showing that the neck-mimic can also amplify more efficiently the plus-end biased conformational changes that are proposed to occur in the kinesin-14’s catalytic core, when the C-terminal neck-mimic anchors the catalytic motor core to the substrate.

**Table 1:** Summary of direction and velocity of microtubule gliding driven by monomeric kinesins.

Kinesin	Construct	Directionality	Velocity [nm s^−1^]
14	BP-Ncd325	− (102/103)^a^	^b^− 5 ± 1^c^(102)
	Ncd325-BP	+ (110/113)	+ 50 ± 9 (110)
	BP-Kar363	− (59/60)	− 2 ± 1 (59)
	Kar363-BP	+ (55/55)	+ 7 ± 2 (55)
	BP-KlpA398	− (62/63)	− 40 ± 9 (62)
	KlpA398-BP	+ (51/52)	+ 64 ± 11 (51)
Mutant	BP-NcdRan14	+ (63/68)	+ 0.4 ± 0.2 (63)
	NcdRan14-BP	+ (51/53)	+ 65 ± 9 (51)
Chimera	^d^BP-nKn664	− (9/9)	− 0.16 ± 0.07 (19)
	nKn664-BP	+ (80/81)	+ 10 ± 1 (80)

The minus (−) and plus (+) sign refer to the minus-end and plus-end polarity, respectively. ^a^Values of denominator in parentheses are the number of microtubules analyzed and values of the numerator are the number of polarity-marked microtubules which moved as indicated (+ or −). ^b^Velocity data is given as mean ± SD. ^c^In assays using either BP-Ncd325, Ncd325-BP, BP-Kar363, Kar363-BP, BP-KlpA398, KlpA398-BP, BP-NcdRan14, NcdRan14-BP or nKn664-BP, of 109, 144, 67, 60, 65, 55, 91, 53 and 84 polarity-marked microtubules in the observation field, 109, 116, 63, 55, 63, 52, 74, 53 and 83 glided. The other 0, 28, 4, 5, 2, 3, 17, 0 and 1 did not glide. Only gliding microtubules that were > 1 µm in length and did not cross each other were analyzed. Values in parentheses are the number of microtubules analyzed. ^d^Results are from Yamagishi et al.^38^.

**Table 2:** Summary of direction and velocity of QD coated with monomeric kinesin-14s along a microtubule.

Kinesin	Construct	Directionality	Velocity [nm s^−1^]
14	BP-Ncd325	−	− 37 ± 16 (171)
	Ncd325-BP	+	+ 64 ± 20 (248)
	BP-Kar363	−	− 17 ± 11 (62)
	Kar363-BP	ND	ND
	BP-KlpA398	−	− 74 ± 26 (100)
	KlpA398-BP	ND	ND
Mutant	BP-NcdRan14	+	+ 10 ± 7 (55)
	NcdRan14-BP	+	+ 69 ± 15 (62)

The minus (−) and plus (+) sign refer to the minus-end-directed and plus-end-directed motility, respectively. In assays using either BP-Ncd325, Ncd325-BP, BP-Kar363, BP-KlpA398, BP-NcdRan14, or NcdRan14-BP, 26, 88, 18, 58, 9 and 19 QDs respectively moved in the indicated direction (+ or −) along polarity-marked microtubules with no exceptions. Only QDs that moved more than ~ 0.25 µm were analysed. Velocity data includes the QD movement along both polarity-marked and non-polarity-marked microtubules and is given as mean ± SD. *n*, number of QDs. ND, not determined. QDs coated with either multiple Kar363-BP or KlpA398-BP molecules did not show unidirectional processive movement of more than 0.25 µm.

### Ncd neck-mimic can replace the kinesin-1 neck-linker in an Ncd—kinesin-1 chimera

To clarify the role of the Ncd neck-mimic, we examined the directionality of a C-terminal anchored Ncd—kinesin-1 chimera nKn664-BP, which contains the kinesin-1 catalytic core with Ncd neck-helix and neck-mimic (Fig. 1b). Previously we had shown that N-linked BP-nKn664 was minus-end-directed^38^, and we now found that C-linked nKn664-BP reversed this to produce a plus-end directionality (Table 1 and Supplementary Fig. 4e). The gliding velocity of 10 nm s^−1^ driven by C-linked nKn664-BP was ~ 50 times faster than that of N-linked BP-nKn664, indicating that the Ncd neck-mimic can replace the kinesin-1 neck-linker in combination with the kinesin-1 catalytic domain. Taken together, these observations suggest that despite their amino acid sequence differences kinesin-1 neck-linker and Ncd neck-mimic are functionally similar. Also that throughout the kinesin superfamily, irrespective of the native directionality of the whole kinesin motor molecule, the kinesin catalytic core itself has a weak mechanical activity directed towards the microtubule plus-end. Furthermore, with the appropriate anchoring of the motor via the C-terminus the small plus-end-directed bias present in the catalytic core can be amplified efficiently by the short C-terminal neck-mimic region to produce rapid motility.

### C-linked kinesin-14 monomers glide microtubules with a left-handed corkscrew like motion

**Figure 4:**
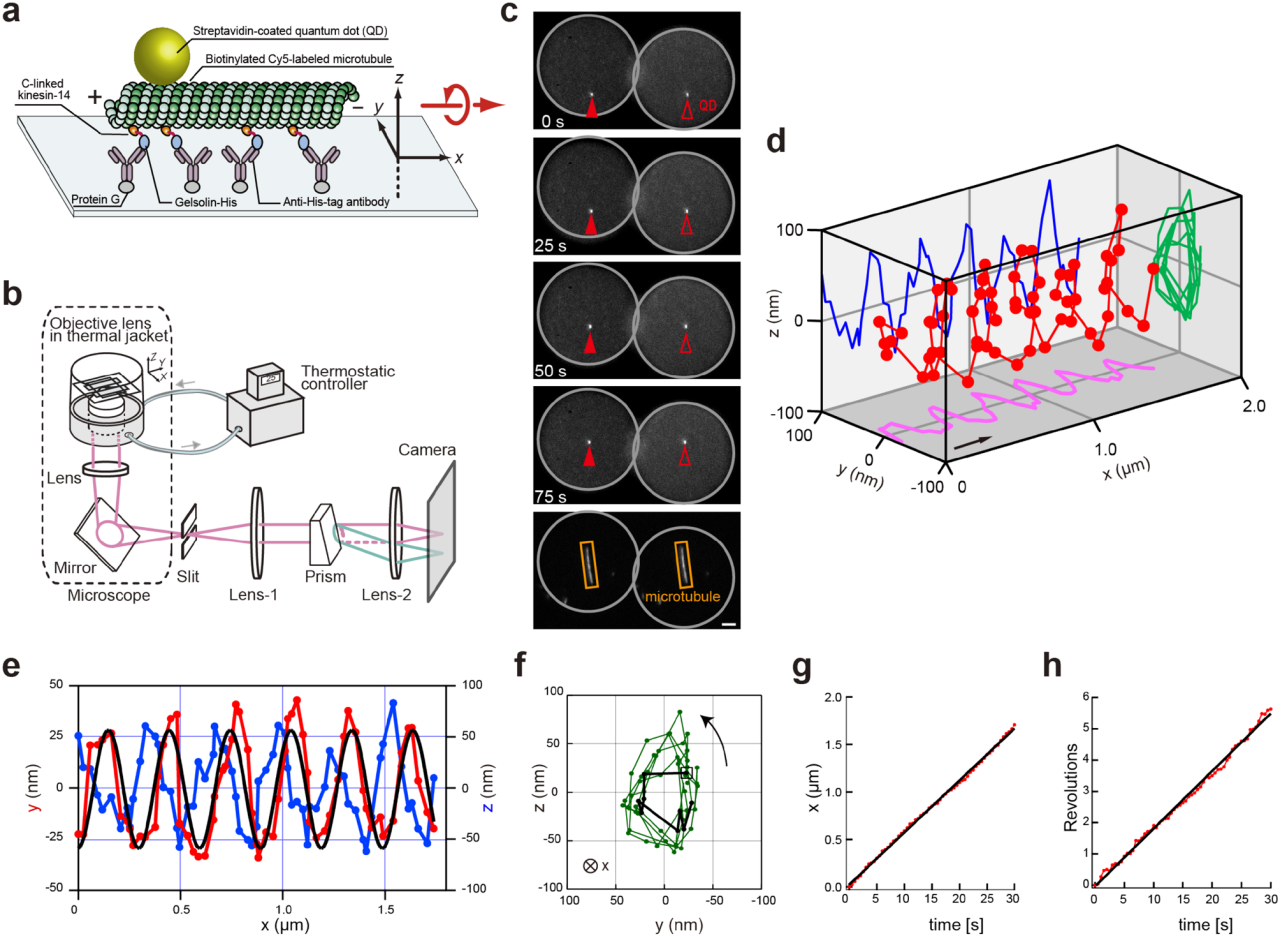
The 3D trajectories of the corkscrew motion of gliding microtubule driven by C-linked Ncd. (**a**) A schematic of the in vitro corkscrewing assay during 3D measurement. The sparsely biotinylated, Cy5-labeled microtubule with a QD (*λ* = 525 nm) attached is corkscrewing driven by C-linked Ncd325-Gel anchored to the Protein G (gray)-coated glass surface via anti-His-tag antibody (purple). (**b**) A Schematic of the *tPOT* setup (not to sale). The *z* position of a QD as well as *x*–*y* position are obtained from a pair of images split by the prism. The temperature in the chamber is maintained at 25 °C by the combined temperature management unit. (**c**) Sequential images of the microtubule-attached QD observed under the *tPOT* microscope. The bottom image shows a Cy5-labeled microtubule, whereas the others show a QD attached to the translocating microtubule. The solid and open arrowheads indicate the images split by the prism of the QD bound to the microtubule, respectively (time in seconds). Scale bar, 2 μm. (**d**) 3D plot of the QD reveals left-handed rotation of the gliding microtubule. The arrow indicates the approximate displacement during 10 s. (**e**) The *x*–*y* (red) and *x*–*z* (blue) trajectories of corkscrewing microtubules driven by Ncd325-Gel shown in (**d**). The rotational pitch of corkscrewing microtubules driven by Ncd325-Gel was determined by fitting the *x*–*y* position of the QD with a sine function (black line), yielding a value of 0.30 µm. (**f**) The *y*–*z* trajectory of the QD bound to the microtubule shown in (**d**). The trajectory of the first revolution is shown by the black line and begins at the open square. The trajectory shows counter-clockwise rotation of the gliding microtubule when looking in the direction of forward translocation. (**g**) Time course of *x*-displacement of the QD bound to the microtubule shown in (**d**). The longitudinal velocity was determined by fitting the *x*–*t* position of the QD with a linear function (black line), yielding a value of 0.055 µm s^−1^. (**h**) Time course of revolutions of the QD bound to the microtubule shown in (**d**). The rotational velocity was determined by fitting the *rev*-*t* position of the QD with a linear function (black line), yielding a value of 0.18 rev s^−1^.

Apart from the processive kinesin-1 dimer^20^, which tracks microtubule protofilaments, most kinesins moving along the microtubule longitudinal axis also generate a torque that causes gliding microtubules in motility assays to rotate around their longitudinal axis. The resulting microtubule corkscrewing motion has been observed in kinesin-1, -2, -3, -5, -6, -8 and -14 constructs. Plus-end-directed N-kinesins cause a left-handed corkscrew^12,14–16,42,49^, whereas minus-end-directed C-kinesin (kinesin-14, dimeric Ncd) cause a right-handed corkscrew^18,19^, implying a similar torque direction relative to the catalytic domain in all kinesins (see Supplementary Fig. 1 for definition of handedness). We therefore examined the microtubule rotation caused by our constructs in microtubule motility assays observed using a three-dimensional prismatic optical tracking (*tPOT*) microscope, which can detect 3D movement with nm accuracy^15^. In motility assays using either Ncd325-Gel, Kar363-Gel or KlpA398-Gel kinesin-14s, which are anchored to the surface via a C-terminal gelsolin fusion and have plus-end-directed motility (Fig. 4a–c and Supplementary Movie 3), gliding microtubules rotated with a left-handed corkscrew of ~ 0.3 μm pitch (Fig. 4d–h and Supplementary Figs. 6 and 7). Thus, in kinesin-14 constructs where the longitudinal directionality has switched from minus to plus ended, the microtubule rotation has also switched from a right-handed to left-handed corkscrewing motion, consistent with the torque direction remaining fixed relative to the kinesin head, despite the reversal of longitudinal direction. Furthermore, the ~ 0.3 μm pitch of the corkscrew is similar to the pitch reported for plus-end-directed kinesins-1, and -5 monomers^15,25^. This result is consistent with the kinesin motor domain having a lateral component of movement with the same magnitude and direction during both plus-end- and minus-end-directed motility (Supplementary Fig. 1). The similarity of pitch, despite differences in velocity, suggests a tight linkage between the conformational changes generating longitudinal motion and torque. It also strongly suggests that the neck-linker of N-kinesins and the neck-mimic of C-kinesins function in the same way to generate the same directional vectors of longitudinal and rotational forces, with the same constant ratio of the two forces in both cases, resulting in the same corkscrew pitch.

## Discussion

The determinants of kinesin motor directionality on microtubules are of considerable interest in understanding the molecular mechanism of kinesin motility. Although previous studies have identified the neck-helix and neck-mimic regions external to the catalytic core that are required for minus-end motility of kinesins-14, the contribution of the N-terminal anchoring arrangement of these motors was less clear. Endow and Waligora found that a dimeric construct NcdKHC1 where the motor domain of kinesin-1 is substituted in place of the Ncd motor domain, but retains the N-terminal neck-helix and stalk of Ncd together with the Ncd neck-mimic, has minus-end-directed motility, reversing the normal plus-ended motility of the kinesin-1 motor^37^. A corresponding monomeric N-linked chimera, BP-nKn664, also has minus-end directionality^38^. Although these reports suggested that N-terminal anchoring may be important, recent observations have made clear that the anchoring geometry of motors is not an absolute requirement for minus-end-directed motility as some N-terminal motor kinesin-5 have been shown to have context dependent minus-end-directed motility^3,4^. However, there may be specific factors related to the structure of kinesin-5 involved in determining its direction since the same factors that cause direction switching in multiheaded kinesin-5 Cin8^3,4^, do not switch direction in monomeric knesin-5 constructs of Cut7^5^ or Cin8^42^. We therefore systematically tested whether the N-terminal anchoring geometry is an absolute requirement for minus-end-directed motility of kinesin-14 motors. To exclude head interactions, unbound head orientation effects or any effect of the stalk region on direction as observed in KlpA^50^, we used single-headed constructs (Fig. 1). By using native monomeric constructs that differ only in the geometry of anchoring to the substrate, we have shown that the longitudinal direction of monomeric kinesin-14’s motility is then determined by the N- or C-terminal location of the kinesin anchor. Only when the kinesin-14 catalytic core is anchored at its N-terminus through the N-terminal neck-helix does it move towards microtubule minus-ends (Figs. 2 and 3). We found that all three kinesin-14 catalytic cores tested, including KlpA, had the same requirement for N-terminal linkage to generate minus-end motility. In relation to the anchoring geometry effect, native full-length dimeric kinesin-14 KlpA have been found to have the ability to switch direction^9^. When the microtubule binding site in the N-terminal tail of KlpA binds to the same microtubule as the catalytic domain it switches from minus-end to plus-end motility. Suggestively our results show that a change in anchoring geometry can also cause direction reversal in a KlpA single-headed construct containing the KlpA neck-helix, catalytic core and neck-mimic, though it remains to be determined if the reversing mechanisms of our construct and the native full length KlpA are related.

The neck-mimic of kinesin-14 has been proposed to fulfil the role of the neck-linker in other kinesins in motility and regulating ATPase activity^33^, despite them sharing little homology in their amino acid sequences. Previously we have shown that the neck-mimic supported plus-end-directed motility in an N-terminal anchored construct (BP-nKn669) or minus-end-directed motility in a construct with 5 amino acids from the Ncd catalytic core (BP-nKn664)^38^. However, it was not clear if the neck-mimic could support plus-end motility in a C-terminal anchored construct, so we examined its function in C-terminal anchored native kinesin-14 and chimeric kinesin-1 constructs. We found that both in the monomeric kinesin-14 native constructs and in a chimeric monomeric construct with a kinesin-1 catalytic core, the kinesin-14 neck-mimic could replace the neck-linker and support plus-end-directed motility in these C-terminal anchored constructs (Fig. 2). In the C-terminal anchoring configuration both the neck-linker and neck-mimic might both act to amplify the conformational changes in the kinesin and increase microtubule motility (Table 1 and Supplementary Fig. 4). These results support the view that the neck-mimic is functionally equivalent to the neck-linker. However, as shown in this study (Supplementary Fig. 3) and previous work^37,38^ for the neck-mimic and neck-helix to generate minus-end-directed motility in hybrid constructs containing the kinesin-1 motor core, a group of 5 amino acids (AASVN) from the C-terminal end of the kinesin-14 catalytic core and N-terminal anchoring must also be present. Minus-end-directed motility in the chimera (NcdKHC6), which has a group of 5 amino acids (GRRAK) from the C-terminal end of the kinesin-1 catalytic core together with the neck-linker, also requires N-terminal anchoring. All minus-end directed motility for both the neck-linker and neck-mimic chimeras required both the neck-helix and N-terminal anchoring to be present. In this work we show that the N-terminal anchoring via a normal neck-helix linkage to the catalytic core is an absolute requirement for minus-end-directed motility of single-headed kinesin constructs.

Unlike the minus-end-directed motility of single-headed kinesin constructs, plus-end-directed motility has no specific requirement for either N- or C-terminal anchoring, so that BP-NcdRan14 with the neck-helix link to the catalytic domain disrupted by insertion of random amino acids has plus-end-directed motility even when anchored by its N-terminus (Fig. 2h and Supplementary Figs. 3). Thus, with the loss of function of any one of the minus-end-directed motility factors, such as proper functioning of the neck-helix, the kinesin-14 motor becomes plus-end directed. This is consistent with a previous suggestion that the conserved catalytic core of all kinesins may have an inherent plus-end bias^39,51^ with minus-end motility being a gain of function overriding this inherent bias. However, we cannot exclude the possibility that since proper functioning of the neck-helix and neck-mimic of kinesin-14 is required for minus-directed polarity, as demonstrated by several mutant and chimeric constructs such as BP-NcdRun14 (Fig. 2 and Tables 1 and 2) and BP-nKn669^38^ (Supplementary Fig. 3), anchoring at the C-terminus of kinesin-14 might disrupt the correct interaction of the neck-helix and neck-mimic and then induce plus-end-directed polarity via a tilt of the neck-mimic toward the plus-end of microtubules due to steric hindrance. However, further support for at least having conserved conformational changes within the catalytic core comes from the conserved torque component of kinesin motility. Previous studies have shown that despite having different longitudinal motility directions along the microtubule, all kinesins tested have the same directional bias of the torque relative to the kinesin head^12–18^, although due to the way the rotation is defined this is somewhat confusingly expressed as a “change” in rotational direction for plus- and minus-end-directed kinesins. Our study has shown that when the normally minus-end-directed kinesin-14 catalytic core constructs have their directionality reversed to become plus-end-directed by C-terminal anchoring not only does this torque directional bias remain, but the pitch of microtubule rotation it causes in motility assays (Fig. 4 and Supplementary Figs. 6 and 7) is similar to that of the normally plus-end-directed monomeric kinesin-1^15,25^ suggesting a tight and conserved linkage between the longitudinal and transverse components. Our results show that the linkage between torque and longitudinal motility is maintained in the catalytic core of both kinesin-14 and kinesin-1 families of kinesins suggesting a strong conservation of catalytic core function.

Previous work has shown that N-terminal anchoring is required for minus-end-directed motility in chimeras of Ncd and kinesin-1 (Supplementary Fig. 3)^37,38^. Our observations show that in native monomeric kinesins-14 constructs (Ncd, Kar3 and KlpA) the neck-helix, kinesin-14 catalytic core and neck-mimic are insufficient to support minus-end motility unless the construct is also anchored to the substrate via the neck-helix located at the N-terminus. If the constructs are anchored via the C-terminal neck-mimic, they become plus-end-directed. Furthermore, the plus-end-directed speed of C-linked kinesin-14 constructs was always higher than the minus-end-directed speed of N-linked constructs, suggesting the same kinetic bias is present in the catalytic domain of both kinesin-1 and kinesin-14. These observations suggest that the default activity of the highly conserved kinesin catalytic core motor domain in kinesin-1 and kinesn-14 families imparts a plus-end-directed and left-handed corkscrew rotation to microtubules. Furthermore, the similar pitch of the corkscrew motion created by diverse motors anchored at their C-terminus suggests that the mechanism is highly conserved in most kinesins.

## Methods

### Construction of plasmids

Monomeric constructs Ncd325 used 325–700 a.a. of *Drosophila melanogaster* Ncd^18^, Kar363 used 363–729 a.a. of *Saccharomyces cerevisiae* Kar3^45^, and KlpA398 used 398–770 a.a. of *Aspergillus nidulans* KlpA^9^; with either an N-terminal or C-terminal avi-tag nucleotide sequence encoding GLNDIFEAQKIEWHE, which is biotinylated in *Escherichia coli*^46^, were cloned into both the His-pColdIII (Takara, Shiga, Japan) and pET17b vectors (Novagen). Monomeric Ncd mutant construct NcdRan14 with an insertion of 14 random residues GESGAKQGEKGESG between 346 and 347 a.a., which should completely disrupt the neck-helix—motor core interaction, was cloned into either the His-pColdIII vector for C-linked NcdRan14 or pET17b vector for N-linked NcdRan14, with a C- or N-terminal avi-tag. For monomeric chimeric kinesin nKn664-BP, nKn664 (comprising DmNcd K325-N348—*Rattus norvegicus* KIF5C I9-K320—DmNcd A664-K700)^38^ was cloned into the His-pColdIII vector with a C-terminal avi-tag. Either Ncd325, Kar363 or KlpA398 were inserted into the Gelsolin-His-pColdIII vector^42^ to create C-terminal gelsolin fusions. All constructs were verified by DNA sequencing. See Fig. 1b and Supplementary Fig. 3 for details.

### Expression and purification of monomeric kinesins

Expression plasmids for avi-tag-fused monomeric kinesins, Ncd mutant and chimera were transformed into *Escherichia coli* strain BL21 Star (DE3) cells (Invitrogen). After expression in BL21 Star (DE3) cells using either 0.1 mM IPTG (Sigma-Aldrich) for 24 h at 15 °C for His-pColdIII vectors (Takara, Shiga, Japan) or 0.4 mM IPTG for 6 h at 23 °C for pET17b vectors (Novagen), cells were pelleted then re-suspended in lysis buffer (pH 7.4: 80 mM PIPES-KOH, 1 mM MgCl_2_, 1 mM EGTA, 100 µM ATP, 1 mM DTT, 0.1% CHAPS, 0.1% Tween20, 10% glycerol, protease inhibitors, and either 500 mM NaCl for his-tagged proteins or 40 mM NaCl for non-his-tagged proteins) and sonicated for 20 min on ice. Then, the lysate was clarified by centrifugation (20 min, 305,000*g*, 2 °C). Expressed his-tagged proteins were purified by immobilized metal affinity chromatography using a HisTrap HP column (Cytiva) and non-his-tagged proteins by cation exchange chromatography using a Hitrap SP HP column (Cytiva). Pooled fractions were exchanged into a desalting buffer (80 mM NaCl, 20 mM potassium phosphate, 1 mM MgCl_2_, 1 mM DTT, 20 µM ATP, pH 7.4) using a HiTrap Desalting column (Cytiva) and then further purified by affinity binding to polymerised microtubules. Taxol-stabilised microtubules were mixed with purified proteins supplemented with 1 mM AMPPNP and incubated for 15 min at 25 °C. After removing the unbound proteins by centrifugation (20 min, 305,000*g*, 23 °C), the microtubule-bound kinesin was eluted with ATP-containing buffer (10 mM ATP, 10 mM MgCl_2_, 200 mM potassium acetate, 20–80 µM taxol in M buffer [20 mM PIPES-KOH, 4 mM MgCl_2_, 10 mM potassium acetate, 1 mM EGTA, pH 7.4]) by incubation for 10 min at 25 °C. Microtubules were finally removed by centrifugation (20 min, 305,000*g*, 23 °C)^42^. Bacterial-expressed monomeric kinesin-14 gelsolin fusions were purified using taxol-stabilized microtubule affinity as described^52^. Purified kinesins were flash frozen and stored in liquid nitrogen. The concentrations of kinesin-14s, mutants or chimeras were estimated by SDS-PAGE on 10% acrylamide gels using BSA standards (Thermo Fisher Scientific) loaded on the same gel^53^. Gels were stained with Quick-CBB PLUS (Wako, Osaka, Japan) and imaged using a CCD camera (CSFX36BC3, Toshiba-teli, Tokyo, Japan). The bands containing kinesins and BSA standards were quantified using ImageJ (NIH).

### Purification of tubulin

Tubulin was purified from porcine brain through four cycles of temperature regulated polymerization and depolymerization in a high molarity PIPES buffer to remove contaminating microtubule-associated proteins^54^. Purified tubulin was flash frozen and stored in liquid nitrogen. Porcine brains were obtained from dead animals and were provided by Shibaura Organ (Tokyo, Japan), an authorized supplier of animal organs.

### Polarity-marked microtubule gliding assays

Cy5-labeled polarity-marked microtubules were prepared as described^38^. First, bright short microtubules (labeled tubulin: unlabeled tubulin = 1 : 2) were polymerized with 0.5 mM GMPCPP (a nonhydrolyzable GTP analog, Jena Bioscience), then plus-end elongation of dim long segments (labeled tubulin: unlabeled tubulin = 1 : 9) was achieved by inclusion of NEM treated tubulin which inhibits minus-end polymerisation. Microtubule gliding assays were performed in flow chambers assembled from KOH-cleaned coverslips attached using double-sided tape (NW-25, Nichiban, Tokyo, Japan or Scotch W-12, 3M) as described^38^. For assays using avi-tag-fused monomeric kinesins, 1 flow chamber volume of 5 mg mL^−1^ biotinylated-BSA (Sigma-Aldrich) was introduced into the flow chamber, incubated for 5 min and then rinsed with 4 volumes of M buffer. The chamber surface was coated sequentially with 1 volume of 5 mg mL^−1^ streptavidin (Wako, Osaka, Japan) for 10 min, 4 volumes of 5 mg mL^−1^ BSA (Sigma-Aldrich) for 3 min and 1 volume of either 0.5–1 µM N- or C-terminal avi-tag-fused Ncd325, Kar363, KlpA398, NcdRan14 and 1 volume of 1 µM of C-terminal avi-tag-fused nKn664 for 3 min and then 4 volumes of ~ 10 µg mL^−1^ Cy5-labeled polarity-marked microtubules in BRB80 buffer [80 mM PIPES-KOH, 1 mM MgCl_2_, 1 mM EGTA, pH 6.8] containing 0.5 mg mL^−1^ BSA and 20 µM taxol. Finally, 4 volumes of motility buffer (M buffer containing 3 mM ATP (Sigma-Aldrich), 20 µM taxol, ATP regeneration system, the oxygen scavenger system, 1 mM DTT, 0.5 mg mL^−1^ BSA) were applied to the chamber. This procedure tethered monomeric kinesin-14s, Ncd mutant NcdRan14, or chimera to the glass surface via either their C-termini or their N-termini. For lawns of N-linked kinesin-14s or NcdRan14, the neck-helix was coupled to the coverslip via an avi-tag so that the N-terminal neck-helix exerts or transmits the gliding force. In contrast, for lawns of C-linked kinesin-14s, NcdRan14 or chimera, tethering was achieved via the C-terminal neck-mimic and the N-terminal neck-helix was disconnected. All chambers were sealed with nail polish. Assays were carried out at 24 ± 1 °C. Microtubule gliding was observed using a fluorescence microscope (Eclipse Ti, Nikon) with a stable stage (KS-N, Chukousya Seisakujo, Tokyo, Japan) and a stage controller (QT-CM2-35, Chuo Precision Ind., Tokyo, Japan), using illumination from a mercury lump (Intensilight, Nikon), 100 × /1.49 NA, Plan-Apochromat objective lenses (Nikon) and Cy5 filter set (Semrock). Images were recorded by EMCCD camera (iXon X_3_ DU-897E-COO-#BV, Andor Technology). Temperature-control equipment (F-12, Julabo, Germany) was used to suppress positional drift, which is mainly caused by temperature variations. Microtubule gliding velocity was calculated by dividing the distance travelled by the time interval (1–40 s) using the automated tracking software Mark2 (kindly provided by Dr. Ken’ya Furuta, NICT)^55^. Only gliding microtubules that were > 1 µm in length and did not cross each other were analyzed. A fixed bright spot on the cover glass was tracked to distinguish the displacement of microtubules by drift from the microtubule gliding driven by slow motor activity. Directionality and velocities were determined using measurements from at least three independent assays for each construct.

### Quantum dot assays

Quantum dot (QD) assays were performed in flow chambers assembled from hydrophobic silanised coverslips attached using double-sided tape (NW-25, Nichiban, Tokyo, Japan). Avi-tag-fused kinesins and streptavidin-coated QDs (Qdot 525 streptavidin conjugated, Thermo Fisher Scientific) were mixed in a molar ratio of 20 : 1 in M buffer for 30 min on ice. For assays using immobilised polarity-marked and non-polarity microtubules, 1 flow chamber volume of 40 μg mL^−1^ anti-β -tubulin antibody (Santacruz, SAP. 4G5) was introduced into the flow chamber, incubated for 10 min and then rinsed with 4 volumes of M buffer. The chamber surface was coated sequentially with 1 volume of 1% (w/v) Pluronic F-127 (Sigma-Aldrich) for 10 min, 4 volumes of 5 mg mL^−1^ casein for 3 min and 1 volume of ~ 20 μg mL^-1^ Cy5-labeled polarity-marked microtubules, which also included some non-polarity-marked microtubules, in BRB80 buffer with 0.5 mg mL^−1^ casein and 20 µM taxol. Then non-immobilised microtubules were washed out with 4 volumes of M buffer containing 20 µM taxol and 0.5 mg mL^−1^ casein. Finally, 50–200 pM QD coated with multiple kinesins in motility buffer (M buffer containing 3 mM ATP (Sigma-Aldrich), 20 µM taxol, ATP regeneration system, the oxygen scavenger system, 1 mM DTT and 0.5 mg mL^−1^ casein) were applied to the chamber. The concentration of kinesin required for binding to the QD was determined in a preliminary screen to determine conditions that permitted us to observe stable motion of a QD along the microtubule. To achieve this the QD needed to be coated with multiple motors that could act as a team. Since the diameter of the QD is ~ 25 nm, this can accommodate multiple kinesins of 5–10 nm in size bound to it. Chambers were sealed with nail polish. Assays were carried out at 24 ± 1 °C. QDs were observed using a fluorescence microscope (Eclipse Ti-PFS, Nikon) with a stable stage (KS-N, Chukousya Seisakujo, Tokyo, Japan) and stage controller (QT-CM2-35, Chuo Precision Ind., Tokyo, Japan), using illumination from a mercury lamp (Intensilight C-HGFIE, Nikon), a 100×/1.49 NA, Plan-Apochromat objective lenses (Nikon) and either a Cy5 filter set for Cy5-labeled microtubules, or a Chroma Qdot 525 set for QDs. Images were recorded by EMCCD camera (iXon X_3_ DU-897E-COO-#BV, Andor Technology). Temperature-control equipment (F-12, Julabo, Germany) was used to suppress positional drift, which is mainly caused by temperature variations^38^. We analyzed the QDs that showed a stable processive motion (> 0.25 μm) along a microtubule. Those on fluctuating, bundled, or crossed microtubules were ignored. QDs that encountered other QDs were also excluded. Individual transporting velocity of each QD covered with multiple monomeric kinesins was calculated by dividing the distance travelled by the time interval (0.3–2 s) using the automated tracking software Mark2^55^. When the QD stopped moving, the analysis was ceased at that point. Fluorescence images of QDs were fitted with a 2D Gaussian to locate the position of the intensity peak of fluorescence, corresponding to the center of the QD. Directionality and velocities were determined using measurements from at least three independent assays for each construct.

### Microtubule corkscrewing assays

Microtubule corkscrewing assays were performed using QD-coated microtubules as described previously^25^. Biotinylated (biotin-(AC5)2 Sulfo-OSu, Dojindo, Kumamoto, Japan), Cy5-labeled (Cy5 Mono NHS Ester, Cytiva) microtubules were prepared by co-polymerizing biotinylated, Cy5-labeled and non-fluorescent tubulin in a molar ratio of 1:3:75 in BRB80 with 1 mM GTP and 1 mM MgCl_2_ for 30 min at 37 °C and then stabilized by 20 μM taxol. QD-coated microtubules were prepared by adding 13 nM streptavidin-coated QD (Qdot 525 streptavidin conjugate, Thermo Fisher Scientific) diluted in BRB80 containing 20 µM taxol (BRB80T) and incubated for 30 min with an equal volume of 4 mg mL^−1^ microtubules and then diluted to 33 pM QD and 10 μg mL^−1^ microtubules in BRB80T containing 0.4 mg mL^−1^ α-casein (Sigma-Aldrich). Microtubule corkscrewing assays were performed in flow chambers assembled from KOH-cleaned coverslips attached using double-sided tape (NW-25, Nichiban, Tokyo, Japan) as described. For assays using His-tag and gelsolin-fused monomeric kinesins, 1 flow chamber volume of 5 mg mL^−1^ Protein G (Sigma-Aldrich) was introduced into the flow chamber, incubated for 5 min and then rinsed with 4 volumes of M buffer. The chamber surface was coated sequentially with 1 volume of 50 μg mL^−1^ anti-His-tag antibody (Qiagen) for 10 min, 4 volumes of 0.4 mg mL^−1^ α-casein for 3 min and 1 volume of either 1 µM C-terminal His-tag-fused Ncd325-Gel, Kar363-Gel or KlpA398-Gel for 5 min and then 4 volumes of ~ 10 µg mL^−1^ Cy5-labeled QD-coated microtubules in BRB80 containing 0.4 mg mL^−1^ α-casein and 20 µM taxol. Finally, 4 volumes of motility buffer (M buffer containing 3 mM ATP (Sigma-Aldrich), 20 µM taxol, ATP regeneration system, the oxygen scavenger system, 1 mM DTT, and 0.4 mg mL^−1^ α-casein) were applied to the chamber. All chambers were sealed with nail polish. Assays were carried out at 25 °C. Temperature-control equipment (F-12, Julabo, Germany) was used to suppress positional drift, which is mainly caused by temperature variations. The principle of 3D tracking has been described previously^15^. The fluorescence was passed through an appropriate filter set (Cy5 filter set, Semrock, for Cy5-labeled microtubules and Qdot 525 set, Chroma, for quantum dots 525 nm). An achromatic lens-1 was placed outside the camera port of the microscope to make an equivalent back focal plane where a custom-made wedge-prism (86.5°) coated with an antireflective layer was precisely set. Two split images of the sample were focused on the camera with another achromatic lens-2 having a desirable *f*, which determined the magnification of the optical system. For calibration of the *z*-axis position in which the objective was displaced at a constant speed, the stable stage was equipped with a pulse motor (SGSP-13ACT, SIGMAKOKI, Tokyo, Japan) and a controller (SHOT-204MS, SIGMAKOKI, Tokyo, Japan). QDs were observed using a fluorescence microscope (Eclipse Ti-PFS, Nikon) with a stable stage (KS-N, Chukousya Seisakujo, Tokyo, Japan) and stage controller (QT-CM2-35, Chuo Precision Ind., Tokyo, Japan), using illumination from a mercury lamp (Intensilight C-HGFIE, Nikon), a 100×/1.49 NA, Plan-Apochromat objective lenses (Nikon) and either a Cy5 filter set for Cy5-labelled microtubules, or a Chroma Qdot 525 set for QDs. Images were recorded at 0.5–1 s intervals by EMCCD camera (iXon X_3_ DU-897E-COO-#BV, Andor Technology). QD movement was tracked and quantified with Igor Pro 5.05A^15^. Handedness and pitch were determined using measurements from at least three independent assays for each construct.

### Statistics and reproducibility

Tracking data for microtubules and QDs were obtained from at least 3 independent experiments, and sample sizes are indicated in detail in the text or the Method section.

### Reporting summary

Further information on research design is available in the Nature Research Reporting Summary linked to this article.

## Supplementary Information

Supplementary Information 1.

Supplementary Information 2.

Supplementary Video 1.

Supplementary Video 2.

Supplementary Video 3.

## Data Availability

All plasmids used in this study are available from the corresponding authors on reasonable request. The source data from this study used to prepare the graphs and tables are provided as Supplementary Data 1.

## Code availability

Custom scripts were written for tracking QDs bound to gliding microtubules with Igor Pro 5.05A. The scripts are available from the corresponding author on reasonable request.
