# Supplementary material for: Anchoring geometry is a significant factor in determining the direction of kinesin-14 motility on microtubules
Source: Sci Rep. 2022 Sep 14;12:15417. doi: 10.1038/s41598-022-19589-4 (PMC9474454; doi:10.1038/s41598-022-19589-4)
Supplement: Supplementary file 1 — Supplementary Information 1. [file 41598_2022_19589_MOESM1_ESM.pdf]

Supplementary Information for

Anchoring geometry is a significant factor in determining the direction of Kinesin-14 motility on microtubules

Masahiko Yamagishi [1], Rieko Sumiyoshi [1], Douglas R. Drummond [2,3], & Junichiro Yajima [1,4,5,6]*

[1] *Department of Life Sciences, Graduate School of Arts and Sciences, The University of Tokyo, 3-8-1 Komaba, Meguro-ku, Tokyo 153-8902, Japan*

[2] *Centre for Promotion of International Education and Research, Faculty of Agriculture, Kyushu University, 744 Motooka, Nishi-ku, Fukuoka 819-0395, Japan*

[3] *School of Interdisciplinary Science and Innovation, Kyushu University, 744 Motooka, Nishi-ku, Fukuoka 819-0395, Japan*

[4] *Komaba Institute for Science, The University of Tokyo, 3-8-1 Komaba, Meguro-ku, Tokyo 153-8902, Japan*

[5] *Research Center for Complex Systems Biology, The University of Tokyo, 3-8-1 Komaba, Meguro-ku, Tokyo 153-8902, Japan*

[6] *Universal Biological Institute, The University of Tokyo, Bunkyo-ku, Tokyo 113-0033, Japan*

**Correspondence should be addressed to J.Y. (yajima@bio.c.u-tokyo.ac.jp)*

Supplementary figures and movies:

Supplementary Figure 1 Directionality of kinesins in previous studies.

Supplementary Figure 2 Kinesin-1 – Ncd chimeras used for determination of directionality in previous studies.

Supplementary Figure 3 The scheme and alignment of functional domains.

Supplementary Figure 4 Microtubule gliding velocities driven by N- or C-linked monomeric kinesin-14s.

Supplementary Figure 5 Transporting velocities of QD coated with N-or C-linked monomeric kinesin-14, Kar363, KlpA398 and NcdRan14.

Supplementary Figure 6 Time course of longitudinal and rotational motion of corkscrewing microtubule driven by C-linked kinesin-14, Ncd325-Gel.

Supplementary Figure 7 The 3D trajectories of the corkscrewing microtubule driven by C-linked kinesin-14s, Kar363-Gel and KlpA398-Gel.

Supplementary Movie 1 Polarity-marked microtubule gliding driven by N- or C-linked monomeric kinesin-14s.

Supplementary Movie 2 QD motility on microtubules driven by N- or C-linked monomeric kinesin-14s.

Supplementary Movie 3 Microtubule corkscrewing motion driven by C-linked monomeric kinesin-14, Ncd325-Gel.

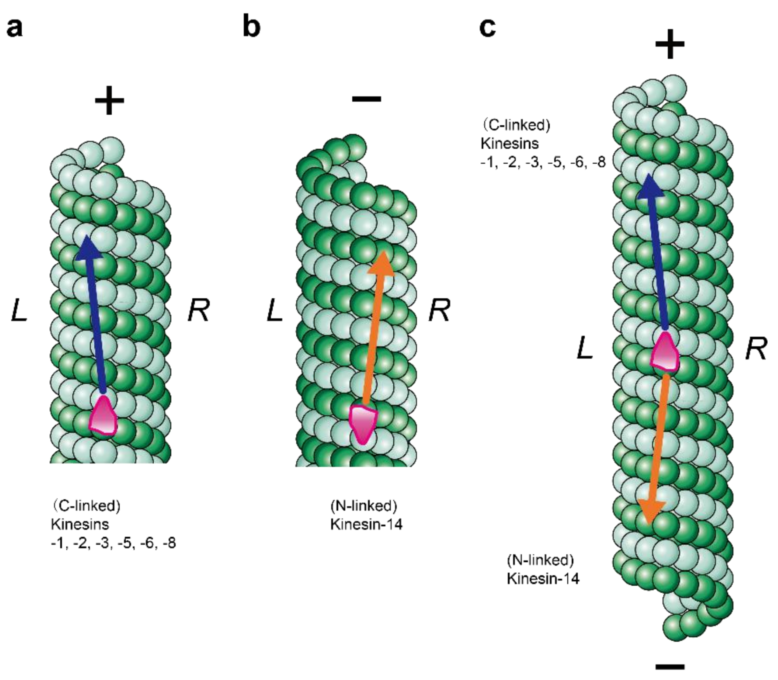

Supplementary Figure 1 Directionality of kinesins in previous studies. N-kinesins such as kinesins-1, -2, -3, -5, -6, -8 are generally found to have plus-endwards and leftwards movements along microtubules (blue arrow) **(a)**, whilst C-kinesins such as kinesin-14 have minus-endwards and rightwards movements along microtubules (orange arrow) **(b)**, when observed from above and the motors are moving away from the observer. These directional movements cause the motors (or team of motors) to follow helical trajectories, when the microtubule is suspended away from the surface, and the motors can move freely all around the entire surface of the microtubule lattice. For example, a double-headed kinesin-1 mutant with a longer neck-linker[1], double-headed kinesin-2 [1], -6 [2], -8 [3] and single-headed kinesin-1 [4], -3 [4], -6 [2] have all been reported to display left-handed helical trajectories, whilst double-headed kinesin-14 displayed right-handed helical trajectories [5]. Direction of blue- and orange-arrows indicate the directions of average stepping of N-kinesins and C-kinesins along the microtubule, respectively. In a microtubule corkscrewing assay, where microtubules move across an array of motors fixed to the surface, motors glide and rotate microtubule (i.e., a corkscrew like motion of microtubule). For example, single-headed kinesins-1 [6,7], -3 [4,8], -5 [7,9], -6 [2] and double-headed kinesin-2 [10], -5 [7], -6 [2], -8 [3,11,12] have been reported to cause left-handed corkscrewing motions of microtubules (microtubule rotates anti-clockwise as it moves away from an observer), whilst double-headed kinesin-14 causes right-handed corkscrewing motion (microtubule rotates clockwise as it moves away from an observer)[13–15]. In both cases if the kinesin motor retains the same orientation with respect to the microtubule plus and minus ends (shown in pink with pointed end always facing the microtubule plus end), then these results imply that the lateral component of the kinesin movement is in the same direction, relative to the kinesin head, for both the plus and minus end directed motors (to the left with the microtubule plus end orientated at the top as drawn in the figure) and only the longitudinal direction has changed **(c)**. Double-headed kinesin-1 can drive microtubule corkscrewing but the handedness and pitch of the microtubule corkscrewing motion depends on the microtubule geometry[12,16–18] since processive kinesin-1 precisely tracks an individual protofilament in the microtubule.

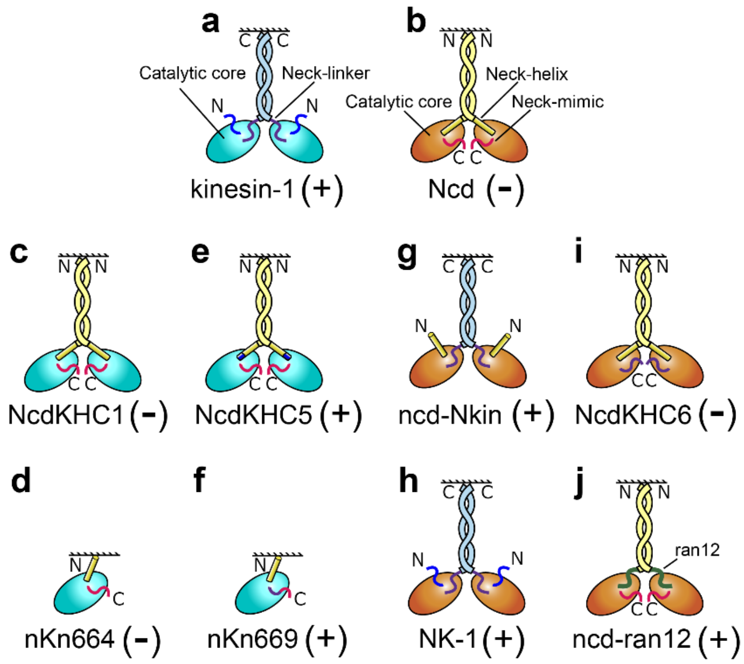

Supplementary Figure 2 Kinesin-1 – Ncd chimeras and Ncd mutant used for determination of directionality in previous studies. **(a)** Kinesin-1 consists of its catalytic motor core (cyan), a neck-linker (purple), a dimerising coiled-coil region (sky blue cord) and an N-terminal cover-strand (blue); and shows plus-end directionality. **(b)** Kinesin-14 Ncd consists of its catalytic motor core (orange), which is ~40 % identical in amino acid sequence to the catalytic motor core of kinesin-1, a neck-helix (yellow cylindrical column), a dimerising coiled-coil region (yellow cord) and a C-terminal neck-mimic region (magenta); and shows minus-end directionality. **(c)** Chimeric NcdKHC1 [19] consists of Ncd neck-helix, *Drosophila* kinesin-1 catalytic motor core, Ncd dimerising coiled-coil region and Ncd neck-mimic. NcdKHC1 shows minus-end directionality, reversing the polarity of kinesin-1 movement. **(d)** Chimeric nKn664 [20] is the monomeric version of dimeric NcdKHC1. nKn664 consists of Ncd neck-helix in the absence of the Ncd dimerising coiled-coil region, rat kinesin-1 catalytic motor core, and Ncd neck-mimic and shows minus-end directionality, reversing the polarity of kinesin-1 movement. **(e)** Chimeric mutant NcdKHC5 [19], which differs from NcdKHC1 by two residues, has a mutation in the Ncd neck-helix – catalytic core junction. The two residues (GN) in the junction are replaced by residues (DS) of the kinesin-1 motor core. NcdKHC5 shows plus-end directionality, retaining the polarity of kinesin-1 movement. **(f)** Chimeric nKn669 [20] in which five residues of α6-helix – neck-mimic junction of nKn664 are replaced with those of kinesin-1, shows plus-end directionality, retaining the polarity of kinesin-1 movement. **(g)** Chimeric ncd-Nkin[21] consists of Ncd neck-helix without the dimerising coiled-coil region, Ncd catalytic motor core, *Neurospora crassa* kinesin-1 neck-linker, and kinesin-1 dimerising coiled-coil region. ncd-Nkin shows plus-end directionality, reversing the polarity of Ncd movement. **(h)** Chimeric NK-1 [22] consists of human kinesin-1 cover-strand, Ncd catalytic motor core, kinesin-1 neck-linker, kinesin-1 dimerising coiled-coil region; and shows plus-end directionality, reversing the polarity of Ncd movement. **(i)** Chimeric NcdKHC6 [19] consists of Ncd dimerising coiled-coil region, Ncd neck-helix, Ncd catalytic motor core, and *Drosophila* kinesin-1 neck-linker. NcdKHC6 has the Ncd dimerising coiled-coil region at the N-terminus and lacks the kinesin-1 dimerising coiled-coil

region at the C-terminus, whilst ncd-Nkin has the kinesin-1 dimerising coiled-coil region at the C-terminus and lacks the Ncd dimerising coiled-coil region at the N-terminus. ncd-Nkin displays plus-end directionality, reversing the polarity of Ncd movement, whilst NcdKHC6 shows minus-end directionality. These results suggested that sufficient neck region, or the dimerising region attached to the catalytic core or neck-linker might determine the motor directionality, rather than the neck-helix–catalytic core junction; however, this has not been tested in previous studies. **(j)** Mutant ncd-ran12 [23] is a two-headed Ncd mutant, in which the neck-helix was mutated by randomizing 12 residues (green). It shows plus-end-directed motility, reversing the polarity of Ncd movement. The plus (+) and minus (-) sign refer to the plus-end and minus-end polarity, respectively.

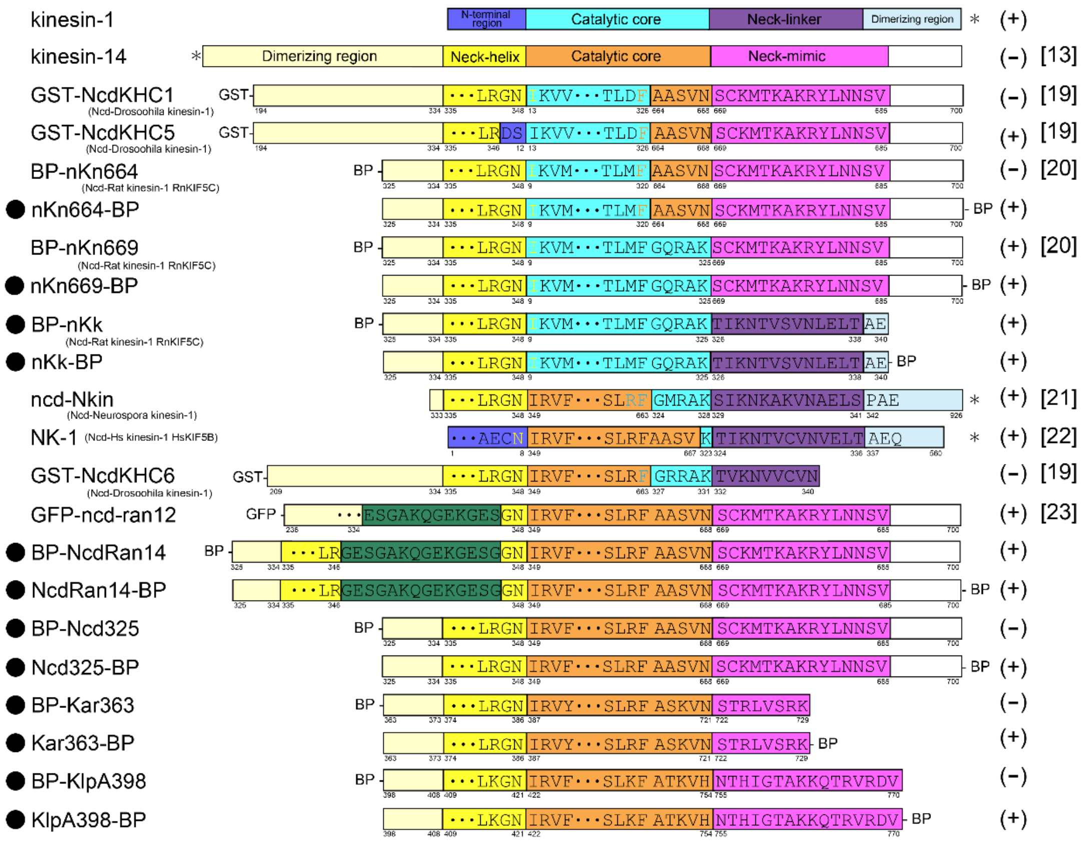

Supplementary Figure 3 The scheme and alignment of functional domains. Schematic representation of chimeric kinesin-1 – Ncd constructs, Ncd mutants, and kinesin-14s used in either previous studies[19–23] or in this study (indicated by filled circles on left of name) to identify determinants for the direction of kinesin movement. Functional domains of kinesin-1 from *Drosophila* (NcdKHC1, NcdKHC5 and NcdKHC6), rat (nKn664, nKn669 and nKk), *Neurospora crassa* (ncd-Nkin), human (NK-1); and kinesin-14 from *Drosophila melanogaster* Ncd325, *Saccharomyces cerevisiae* Kar363, and *Aspergillus nidulans* KlpA398, are aligned and colored. In motility assays, NcdKHC1, NcdKHC5 and NcdKHC6 could be attached to the glass surface via an N-terminal GST-tag[19]. Two constructs were created for each of Ncd325, Kar363, KlpA398, nKn664, nKn669, nKk, and NcdRan14, with either an N- or C-terminal avi-tag (labelled BP) for anchoring them to the glass surface. A recent study from our group reported that N-linked BP-nKn664 and BP-nKn669 were minus- and plus-end-directed, respectively[20]. In this study, we examined the directionality of C-linked nKn664-BP and nKn669-BP. C-linked nKn664-BP reversed minus-end directionality of N-linked BP-nKn664, indicating that minus-end directionality of nKn664 requires tethering the N-terminal neck-helix to the surface. We also made another chimera, nKk (DmNcd K325-N348 – RnKIF5C I9-K340), and examined its directionality. ncd-Nkin[21] and NK-1 [22] were attached to the surface via a C-terminal tail domain of kinesin-1 that has been reported to bind to glass surfaces (asterisk). ncd-ran12 could be attached to the glass surface via an N-terminal GFP-tag[23]. At the right border, directionality and references are given. Note that all C-linked kinesins, kinesin mutants and chimeras are plus-end-directed, even if the constructs have the native neck-helix region at the N-terminus. The plus (+) and minus (-) signs refer to the microtubule plus-end and minus-end directionality, respectively.

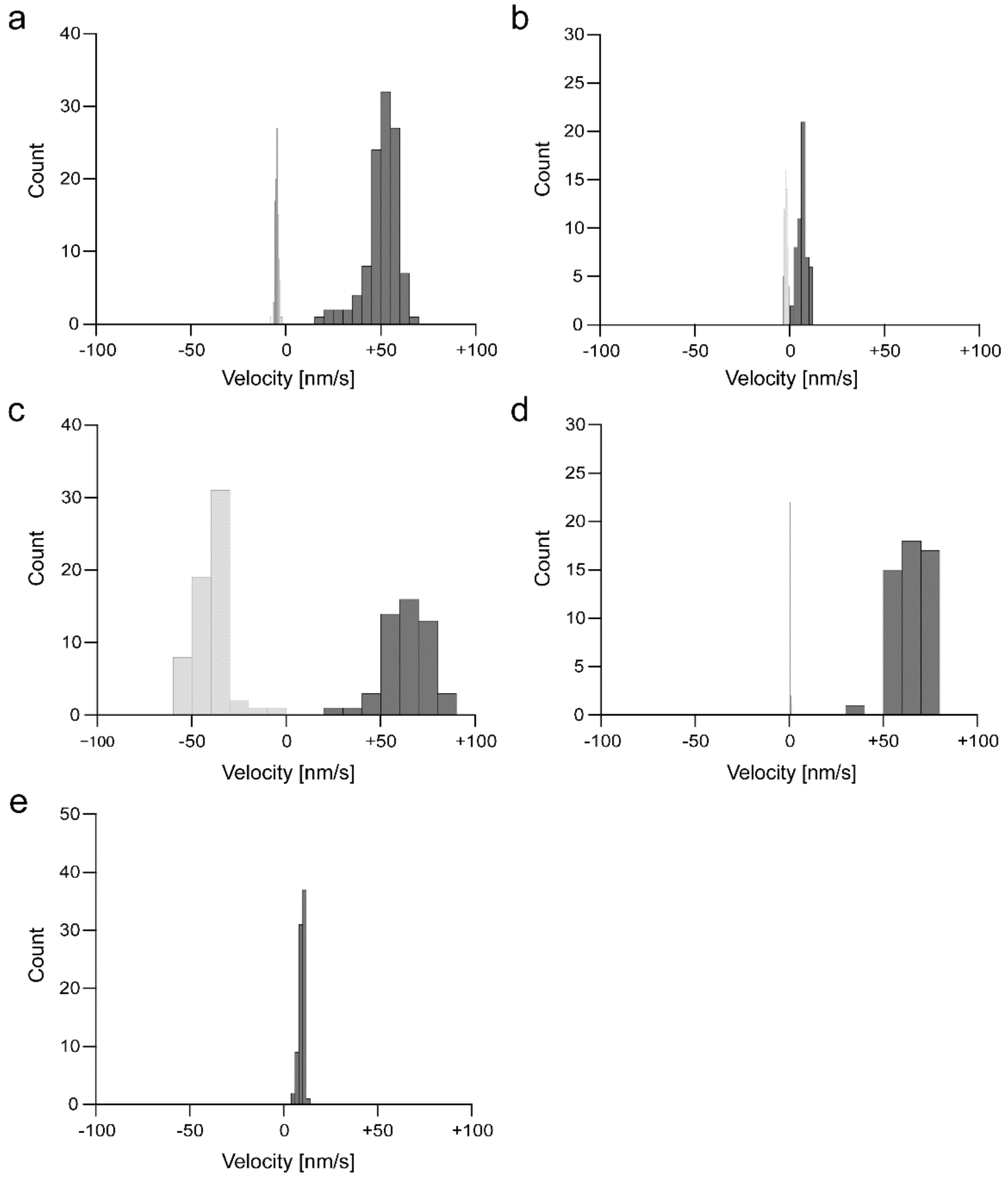

Supplementary Figure 4 A histogram of microtubule gliding velocity driven by N-linked kinesin-14s (gray) and C-linked kinesin-14s (black). **(a)** Ncd325, **(b)** Kar363, **(c)** KlpA398, **(d)** NcdRan14, and **(e)** nKn664 are shown. The plus (+) and minus (-) signs refer to the plus-end-directed motor activity and minus-end-directed motor activity, respectively. Average velocities are shown in Table1.

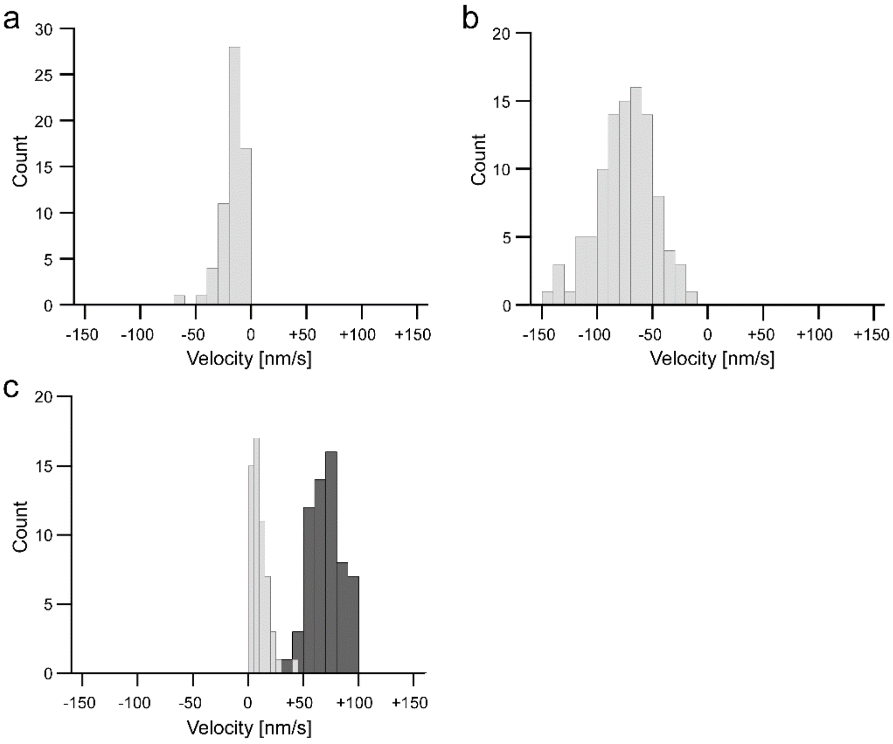

Supplementary Figure 5 A histogram of motility velocities of a QD coated with N-linked monomeric kinesin-14s (gray) and C-linked monomeric kinesin-14s (black). **(a)** Kar363, **(b)** KlpA398, and **(c)** NcdRan14 are shown. Individual traces of QDs moving along microtubules were fitted with linear functions to obtain the longitudinal velocity. The plus (+) and minus (-) signs refer to the plus-end-directed motor activity and minus-end-directed motor activity, respectively. Average longitudinal velocities of QDs are shown in Table 2. QDs coated with either multiple Kar363-BP or KlpA398-BP molecules did not show processive movement of more than 0.25 μm.

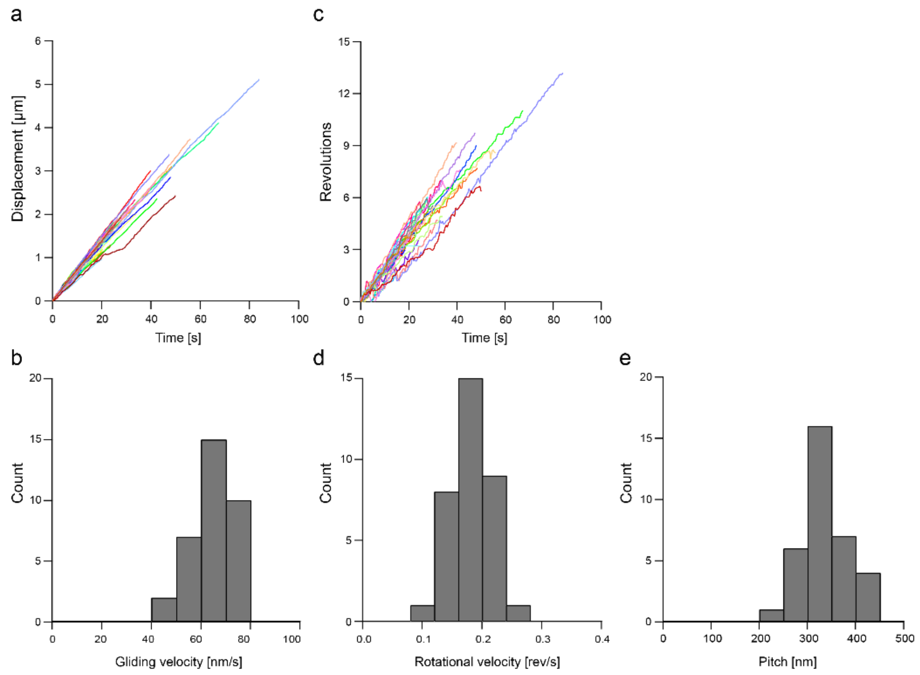

Supplementary Figure 6 Time course of the longitudinal and rotational motions of corkscrewing microtubules driven by C-linked kinesin-14, Ncd325-Gel. **(a)** Time course of longitudinal distances of QDs bound to the microtubule ($n = 34$ microtubules). **(b)** Histogram of the longitudinal gliding velocity. Individual traces in (a) were fitted with linear functions to obtain the longitudinal velocity. Average gliding velocity, 0.065 ± 0.007 µm s^{-1} (mean ± standard deviation (SD), n = 34). **(c)** Time course of revolutions of QDs bound to the microtubules ($n = 34$ microtubules). **(d)** Histogram of the rotational velocity. Individual traces in **(c)** were fitted with linear functions to obtain the rotational velocity. Average rotational velocity, 0.18 ± 0.03 rev s^{-1} (mean ± SD, n = 34). **(e)** Histogram of the corkscrewing pitch. Individual traces of x-y position of the QD were fitted with sin functions to obtain the pitch. Average corkscrewing pitch, 0.33 ± 0.04 µm (mean ± SD, $n = 34$).

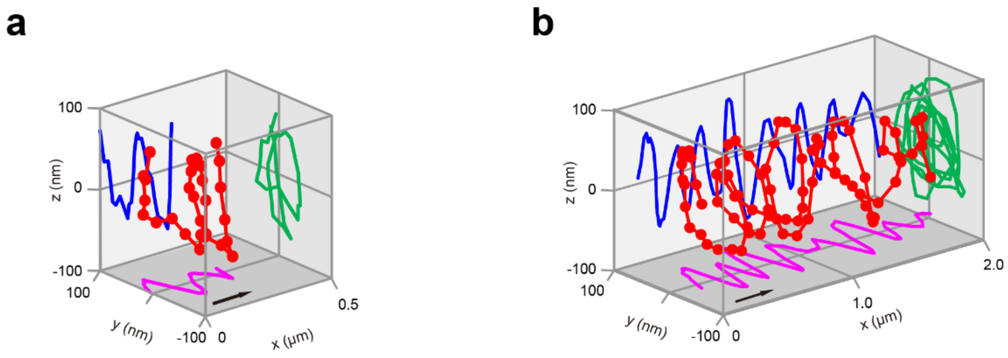

Supplementary Figure 7 The 3D trajectories of the corkscrewing microtubule driven by C-linked kinesin-14s, Kar363-Gel or KlpA398-Gel.

The 3D-plots (red), *x*-*y* (pink), *x*-*z* (blue) and *y*-*z* (green) trajectories of corkscrewing microtubules driven by **(a)** Kar363-Gel and **(b)** KlpA398-Gel. Images were recorded at 1 s and 0.5 s intervals for Kar363 and KlpA398. The rotational pitch was determined by fitting the *x*-*y* position of the QD with a sine function, yielding a value of 0.28 μm for Kar363-Gel and 0.23 μm for KlpA398-Gel. The corkscrewing motions for both constructs were left-handed. The black arrows indicate the approximate displacement after 10 s. The concentrations of kinesin and ATP applied were 1 μM and 3 mM, respectively.

Supplementary References

1. Brunnbauer, M. *et al.* Torque generation of kinesin motors is governed by the stability of the neck domain. *Molecular Cell* **46**, 147–158 (2012).
2. Maruyama, Y. *et al.* CYK4 relaxes the bias in the off-axis motion by MKLP1 kinesin-6. *Communications Biology* **4**, 180 (2021).
3. Mitra, A., Ruhnow, F., Girardo, S. & Diez, S. Directionally biased sidestepping of Kip3/kinesin-8 is regulated by ATP waiting time and motor–microtubule interaction strength. *Proceedings of the National Academy of Sciences* **115**, 7950–7959 (2018).
4. Sugawa, M., Maruyama, Y., Yamagishi, M., Cross, R. A. & Yajima, J. Torque force in the kinesin stroke drives coupled yaw axis and orbital rotations of kinesin coated gold nanorods. *bioRxiv* 2021.11.07.467662 (2021) doi:10.1101/2021.11.07.467662.
5. Mitra, A. *et al.* Kinesin-14 motors drive a right-handed helical motion of antiparallel microtubules around each other. *Nature Communications* **11**, 2565 (2020).
6. Yajima, J. & Cross, R. A. A torque component in the kinesin-1 power stroke. *Nature Chemical Biology* **1**, 338–341 (2005).
7. Yajima, J., Mizutani, K. & Nishizaka, T. A torque component present in mitotic kinesin Eg5 revealed by three-dimensional tracking. *Nature Structural & Molecular Biology* **15**, 1119–1121 (2008).
8. Mitra, A. *et al.* A Brownian Ratchet Model Explains the Biased Sidestepping of Single-Headed Kinesin-3 KIF1A. *Biophysical Journal* **116**, 2266–2274 (2019).
9. Yamagishi, M., Maruyama, Y., Sugawa, M. & Yajima, J. Characterization of the motility of monomeric kinesin-5/Cin8. *Biochemical and Biophysical Research Communications* **555**, 115–120 (2021).
10. Pan, X., Acar, S. & Scholey, J. M. Torque generation by one of the motor subunits of heterotrimeric kinesin-2. *Biochemical and Biophysical Research Communications* **401**, 53–57 (2010).
11. Bormuth, V. *et al.* The highly processive kinesin-8, Kip3, switches microtubule protofilaments with a bias toward the left. *Biophysical Journal* **103**, L4-6 (2012).
12. Mitra, A., Ruhnow, F., Nitzsche, B. & Diez, S. Impact-free measurement of microtubule rotations on kinesin and cytoplasmic-dynein coated surfaces. *PLoS ONE* **10**, 1–18 (2015).
13. Walker, R. A., Salmon, E. D. & Endow, S. A. The Drosophila claret segregation protein is a minus-end directed motor molecule. *Nature* **347**, 780–2 (1990).
14. Nitzsche, B. *et al.* Working stroke of the kinesin-14, ncd, comprises two substeps of different direction. *Proceedings of the National Academy of Sciences* **113**, 6582–6589 (2016).
15. Chandra, R., Salmon, E. D., Erickson, H. P., Lockhart, A. & Endow, S. A. Structural and functional domains of the Drosophila ncd microtubule motor protein. *The Journal of Biological Chemistry* **268**, 9005–9013 (1993).
16. Ray, S., Meyhöfer, E., Milligan, R. A. & Howard, J. Kinesin follows the microtubule's protofilament axis. *The Journal of Cell Biology* **121**, 1083–1093 (1993).
17. Nitzsche, B., Ruhnow, F. & Diez, S. Quantum-dot-assisted characterization of microtubule rotations during cargo transport. *Nature Nanotechnology* **3**, 552–556 (2008).
18. Bugiel, M., Mitra, A., Girardo, S., Diez, S. & Schäffer, E. Measuring Microtubule Supertwist and Defects by

Three-Dimensional-Force-Clamp Tracking of Single Kinesin-1 Motors. *Nano Letters* **18**, 1290–1295 (2018).

19. Endow, S. A. & Waligora, K. W. Determinants of kinesin motor polarity. *Science* **281**, 1200–2 (1998).
20. Yamagishi, M. *et al.* Structural basis of backwards motion in kinesin-1-kinesin-14 chimera: Implication for kinesin-14 motility. *Structure* **24**, 1322–1334 (2016).
21. Henningsen, U. & Schliwa, M. Reversal in the direction of movement of a molecular motor. *Nature* **389**, 93–6 (1997).
22. Case, R. B., Pierce, D. W., Hom-Booher, N., Hart, C. L. & Vale, R. D. The Directional Preference of Kinesin Motors Is Specified by an Element outside of the Motor Catalytic Domain. *Cell* **90**, 959–966 (1997).
23. Sablin, E. P. *et al.* Direction determination in the minus-end-directed kinesin motor ncd. *Nature* **395**, 813–6 (1998).

Movie Legends

Supplementary Movie 1 Polarity-marked microtubule gliding driven by N- or C-linked monomeric kinesins. N-linked monomeric kinesin-14s; BP-Ncd325 (a), BP-Kar363 (c), BP-KlpA398 (e) glide microtubules with their plus ends leading, indicating a minus-end-directed motor activity, whereas C-linked monomeric kinesin-14s; Ncd235-BP (b), Kar363-BP (d), KlpA398-BP (f), N- and C-linked NcdRan14 (g and h) glide with their minus ends leading, indicating a plus-end-directed motor activity. These movies are 5 seconds long at ×100 (a), ×14 (b), ×160 (c), ×60 (d), ×24 (e), ×12 (f), ×400 (g) and ×10 (h) speed, respectively. Each image shows a region 14 × 14 μm. Scale bar: 2 μm.

Supplementary Movie 2 QD motility on microtubules driven by N- or C-linked monomeric kinesin-14s. QDs coated with N-linked monomeric BP-Ncd325s(a), BP-Kar363s (c) and BP-KlpA398s (d) move towards the bright-minus-end of a polarity-marked microtubule, indicating a minus-end-directed motor activity, whereas QDs with C-linked monomeric Ncd325-BPs (b) move towards the dim-plus-end of a polarity-marked microtubule, indicating a plus-end-directed motor activity. Both N- and C-linked monomeric Ncd mutant, NcdRan14 move towards the dim-plus-end of a polarity-marked microtubule, indicating a plus-end-directed motor activity (e and f). The plus (+) and minus (-) signs refer to the plus-end and minus-end of microtubules, respectively. These movies are 4 seconds long at ×20 speed. Each image shows a region 14 × 7 μm. Scale bar: 2 μm.

Supplementary Movie 3 Microtubule corkscrewing motion driven by C-linked monomeric kinesin-14, Ncd325-Gel. The movement of a QD fixed to a microtubule being glided by C-linked Ncd325-Gel was imaged. The movie shows the field of views split by a prism using the *tPOT* system, for ~100 seconds (0.5 s intervals, ×20 speed). Image shows a region 34 × 19 μm. Scale bar: 2 μm.
